# Supplementary material for: Dopamine and serotonin differentially associated with reward and punishment processes in humans: A systematic review and meta-analysis
Source: JAMA Psychiatry. Author manuscript; Available in PMC 2025 Jul 29. (PMC12159863; doi:10.1001/jamapsychiatry.2025.0839)
Supplement: Supplemental Materials [file EMS206494-supplement-Supplemental_Materials.pdf]

Supplementary Materials

Table of Contents

1 Supplementary Methods 2

1.1 Search strategy 2

1.2 Paper selection 4

1.3 Meta-analysis 5

1.3.1 Risk attitude and model-based learning/flexibility 5

1.3.2 Categorization of dopaminergic agents 6

1.3.3 Assessment of heterogeneity and outliers 7

1.3.4 Assessment of publication bias and moderators 7

1.3.5 Assessment of study quality 7

1.4 Effect size calculations 8

1.4.1 Conversion of outcome measures into effect sizes and associated variances 8

2 Supplementary results 13

2.1 Assessment of study quality 14

2.2 Additional dopamine results 15

2.3 Dopamine heterogeneity 20

2.4 Additional serotonin results 22

2.5 Serotonin heterogeneity 25

2.6 Moderator analyses 27

2.6.1 Dopamine 27

2.6.2 Serotonin 28

2.7 Publication bias 30

2.8 Additional supplementary analyses 32

3 References 39

4 eAppendix 1 50

4.1 PRISMA 2020 Checklist 50

4.2 PRISMA 2020 Abstract Checklist 53

1 Supplementary Methods

1.1 Search strategy

eTable 1 lists our search strategy for each database via Ovid, including the MeSH terms. The search terms were refined on a set of 40 in-lab references[1-40] to ensure they captured all relevant studies.

	**MEDLINE/PubMed**		**Embase**		**PsycInfo**
1	Dopamine/	1	catecholamine/	1	exp catecholamines/
2	exp Dopamine Agents/	2	exp *dopamine receptor stimulating agent/	2	exp dopamine agonists/
3	Serotonin/	3	exp *serotonin receptor affecting agent/	3	exp dopamine antagonists/
4	exp Serotonin Agents/	4	tryptophan/	4	exp Serotonin Agonists/ or exp Serotonin/ or exp Serotonin Antagonists/
5	tryptophan/ or tyrosine/	5	tyrosine/	5	tryptophan/
6	dopamine*.ti,ab.	6	dopamine*.ti,ab.	6	tyrosine/
7	seroton*.ti,ab.	7	seroton*.ti,ab.	7	dopamine*.ti,ab.
8	tryptophan*.ti,ab.	8	tryptophan*.ti,ab.	8	seroton*.ti,ab.
9	tyrosine*.ti,ab.	9	tyrosine*.ti,ab.	9	tryptophan*.ti,ab.
10	1 or 2 or 3 or 4 or 5 or 6 or 7 or 8 or 9	10	1 or 2 or 3 or 4 or 5 or 6 or 7 or 8 or 9	10	tyrosine*.ti,ab.
11	learning/ or avoidance learning/ or reinforcement, psychology/ or punishment/ or reward/ or reversal learning/	11	learning/ or exp *associative learning/ or exp *conditioning/ or exp *"reinforcement (psychology)"/ or exp *reversal learning/ or exp *"reinforcement learning (machine learning)"/	11	1 or 2 or 3 or 4 or 5 or 6 or 7 or 8 or 9 or 10
12	motivation/ or exploratory behavior/ or goals/	12	exp *monetary reward/ or reward seeking behavior/ or reward/	12	learning/ or reward learning/
13	decision making/ or choice behavior/ or delay discounting/ or uncertainty/	13	exp *motivation/ or exploratory behavior/	13	behavior/ or exp approach avoidance/ or exp approach behavior/ or exp avoidance/ or exploratory behavior/
14	(reward* or motivat* or punish* or reinforce* or decision* or effort* or learn* or incentive* or choice*).ti,ab.	14	exp *decision making/ or exp *cognitive bias/	14	computational reinforcement learning/
15	11 or 12 or 13 or 14	15	(reward* or motivat* or punish* or reinforce* or decision* or effort* or learn* or incentive* or choice*).ti,ab.	15	exp reinforcement/
16	(participa* or subject* or individ* or human* or healthy* or investigat* or experiment*).ti,ab.	16	11 or 12 or 13 or 14 or 15	16	exp motivation/
17	research subjects/ or healthy volunteers/ or volunteers/	17	(participa* or subject* or individ* or human* or healthy* or investigat* or experiment*).ti,ab.	17	exp decision making/

18	16 or 17	18	research subject/ or volunteer/ or normal human/	18	exp decision theory/
19	10 and 15 and 18	19	17 or 18	19	exp delay discounting/
20	limit 19 to (english language and "humans only (removes records about animals)")	20	10 and 16 and 19	20	(reward* or motivat* or punish* or reinforce* or decision* or effort* or learn* or incentive* or choice*).ti,ab.
				21	12 or 13 or 14 or 15 or 16 or 17 or 18 or 19 or 20
				22	(participa* or subject* or individ* or human* or healthy* or investigat* or experiment*).ti,ab.
				23	exp experimental subjects/
				24	volunteers/
				25	22 or 23 or 24
				26	11 and 21 and 25
				27	limit 26 to (human and english language)

eTable 1: Complete search and MeSH terms for each database.

37

1.2 Paper selection

Data were requested from authors mainly to obtain sufficient information from within-subject studies to pool together with between-subject studies or to obtain individual data for high and no/low reward conditions in the reward response vigor category. If a study reported data from multiple tasks, we extracted data from the most commonly used task in that category. We also prioritized parameters over model-agnostic measures wherever possible, and outcome measures that were as similar to other outcome measures within the same category.

In cases where multiple drugs or dosages were reported, we chose the more commonly used drug in that category and the highest dosage, respectively. For example, for studies that included both tryptophan depletion and tryptophan loading conditions (N=3), we prioritized the depletion condition, as it was the more commonly used and established manipulation in the literature. This decision also ensured statistical independence, as including both conditions from the same study would introduce duplicate samples.

We excluded studies using drugs that have unclear, complex or substantially mixed dopamine or serotonin targets and/or those that would not be considered typical neuromodulatory targets of antidepressants, including LSD studies. While LSD has shown to have interesting effects on RL[41-43], LSD is considered a novel, experimental antidepressant, which is thought to act differently to traditional antidepressants and shown to act on both serotonin and dopamine receptors[44]. A key aim of this meta-analysis was to 1) focus on research that more directly relates to typical serotonergic and dopaminergic-based antidepressants and 2) examine serotonin and dopamine systems separately. Given the already high heterogeneity in our included studies, we opted to exclude psychedelics like LSD to maintain a clearer mechanistic focus on traditional serotonergic and dopaminergic modulation.

For the reward learning/sensitivity category, we only included measures that related to reward processing and could be dissociated from punishment if those were used and vice versa for the punishment learning/sensitivity category. As our initial aim was to examine RL components, we extracted computational parameters for our outcome measure where possible. For the learning/sensitivity subcomponents, we prioritized learning rate over sensitivity parameters. Sensitivity parameters are harder to interpret, as they capture both sensitivity and nonspecific noisiness. For this reason — and due to the greater number of studies using learning rates — we prioritized learning rates in our main analysis.

For the reward response vigor category, we primarily included measures that compared high-reward conditions to no-reward or low-reward conditions. This approach was intended to

specifically capture the influence of dopamine/serotonin on reward-induced invigoration, rather than general motor functions. If these measures were not extractable however, we included a measure of vigor on high reward only if the effect of drug on the no or low reward condition was nonsignificant (N=2 in the dopamine domain). Excluding these two studies[45,46] slightly reduced the pooled effect size in the reward response vigor subcategory (SMD=0.23; 95% CI [0.01, 0.44]) and the overall reward domain meta-analysis for dopamine (SMD=0.16; 95% CI [0.07, 0.25]) but did not change inference.

For the overall reward/punishment meta-analyses, we excluded any studies with duplicate samples. This issue only arose in the dopamine domain of the overall reward meta-analysis, where two pairs of studies had overlapping samples[38,47,48]. As a conservative approach, we retained the studies with the smallest effect sizes from each pair.

Due to our wide search strategy, a number of studies were excluded based on unsuitable cognitive tasks. Overall, we excluded any tasks that could not be easily categorized within the RL subcomponents outlined in the introduction. Specifically, we excluded: tasks where reward and punishment learning could not be disentangled in the outcome measure (e.g., Iowa Gambling Task); tasks that did not primarily assess a reward- or punishment-based process, such as cognitive control, impulsivity, memory, perceptual, or emotional processing tasks; tasks designed primarily to study social behavior (e.g., Dictator Game, Trust Game) or drug-seeking behavior (e.g., drug-preference tasks); studies that used inappropriate outcome measures, such as those that did not report reward- or punishment-specific learning outcomes or lacked behavioral outcome measures (e.g., some Pavlovian tasks that measured only physiological responses); and tasks that were not comparable to others included in the meta-analysis, or where rewards and punishments were not explicitly used as outcome measures.

Preprints were included but abstracts or unpublished work were not. Articles were independently assessed by A.M. and either Z.Q. or T.E. Conflicts were resolved through in-person discussion with Q.J.M.H. Data were extracted by A.M. and Z.Q. and double-checked by A.M. and Z.Q. or Q.D. or Y.A.

1.3 Meta-analysis

1.3.1 Risk attitude and model-based learning/flexibility

As supplementary analyses, we examined risk attitude and model-based learning/flexibility. The risk attitude component examines how risk averse or risk-seeking individuals are. This component is usually examined with gamble tasks where individuals make a choice between a

gamble of some risk between winning or losing and a sure or lower risk option with lower rewards.

Model-based learning/flexibility were composed of tasks that traditionally examine model-based learning or reversal learning. Model-based learning describes how individuals need to acquire a model of the task to make goal-directed choices, in contrast to model-free learning which relies on trial-and-error learning[49]. Similarly, in reversal learning tasks individuals are exposed to changes in the task rules and have to infer when the context has changed to make appropriate decisions. These tasks are thought to measure flexibility, but also model-based aspects since an understanding of the task rules are required.

Risk attitude was excluded from the main meta-analyses because, while it is categorized as a cost-benefit decision-making process and sometimes grouped with effort and temporal delay discounting, it does not necessarily integrate well with these constructs. For instance, in risk attitude paradigms, impulsivity manifests as greater risk-seeking behavior, which however corresponds to lower reward discounting by risk. In contrast, temporal delay tasks define impulsivity as choosing smaller, sooner rewards over larger, delayed ones, which also reflects higher reward discounting by time. Given these opposing directions in reward discounting when considering impulsivity and the frequent inclusion of both rewards and punishments in gambles in risk attitude tasks, we treated risk attitude as a separate construct.

The model-based learning/flexibility subcomponent was also considered as a separate construct as it contains measures that tap into a more complex reward processing domain and some outcome measures in this task had mixed reward and punishment features. We also examined within-subject studies only of both of these subcomponents since a number of within-subject studies could not be combined with the between-subject studies.

1.3.2 Categorization of dopaminergic agents

For the primary analysis, we categorized low-dose dopamine agonists and antagonists based on their opposing pharmacological effects. This was based on several lines of evidence suggesting that pre- or post-synaptic binding depends on dosage. For example, low dose of D2-receptor antagonists has shown to block presynaptic autoreceptors, which are inhibitory, and therefore increase, instead of block, dopamine transmission[50-52]. Similarly, studies in humans have shown that a low dose antagonist increase ventral striatal response to rewards – a typical response to boosting dopamine[53] – as well as serum prolactin levels[14]. A similar pattern has been observed for some agonists at low dosages, like pramipexole[27].

1.3.3 Assessment of heterogeneity and outliers

Heterogeneity was assessed with τ^2, quantifying the variance of the true effect sizes, and the I^2 statistic, representing the percentage of variability in the effect sizes not accounted for by sampling error[54]. Heterogeneity was interpreted as low (25-50%), medium (50-75%) or high (>75% I^2)[54]. Studies were classified as outliers if their 95% confidence interval did not overlap with the pooled effect CI, using the dmetar package in R[55]. To evaluate the influence of outliers, sensitivity analyses were conducted by recalculating the pooled effect and heterogeneity after removing the outliers.

1.3.4 Assessment of publication bias and moderators

Small-study effects were assessed with the Egger test[56] ($p<0.05$) for categories with at least 10 studies[57]. Exploratory moderator analyses (random-effects categorical or metaregression models in R using the metafor package) explored potential explanations for the between-subject variance of effect sizes. These included 1) drug type (agonist or antagonist), 2) more specific dopamine drug activity types (D2 antagonist, D2 agonist, dopamine precursor or dopamine reuptake inhibitor), 3) task type, 4) dose regimen (single or multiple), 5) mean age across the study and 6) the mean proportion of female participants included in the study. The R^2 value was used to quantify the proportion of between-study heterogeneity accounted for by the moderators.

1.3.5 Assessment of study quality

The quality of studies were assessed with the Cochrane Collaboration's tool for assessing risk of bias[58] independently by Z.Q., Q.D., and T.S.

Assessed criteria:

1. Random sequence generation (selection bias)
2. Allocation concealment (selection bias)
3. Blinding of participants and researchers (performance bias)
4. Blinding of outcome assessment (detection bias)
5. Incomplete outcome data (attrition bias)

For each item, the risk of bias was marked as 'low' if it was explicitly mentioned, 'high' if it was not explicitly mentioned, and 'unclear' if it was not explicitly mentioned but might be true.

1.4 Effect size calculations

The SMDs for between-subject studies were calculated using Cohen's *d*[59,60]. These were extracted from papers reporting the required statistics or by using a plot digitizer (PlotDigitizer, 3.1.6, 2024). Both within- and between-subject designs were included since this design choice is irrelevant to the question and there were a substantial number of each (23 between-subject dopamine studies, 45 within-subject dopamine studies; 25 between-subject serotonin studies, 14 within-subject studies).

However, combining within- and between-subject data in one meta-analysis requires knowledge of the correlation between the drug and placebo condition from within-subject studies in order to calculate an equivalent (correlation-corrected) effect size to the between-subject design Cohen's *d*[61,62]. Since none of the included studies reported this, we only included within-subject studies where we could either derive the correlation from the information provided (i.e., where the mean and SD of the drug and placebo condition, and a t-statistic or standard error of the difference were reported) or data were provided from contacted authors. For these within-subject studies, we calculated the Cohen's d_{rm} and associated variance using the Cousineau and Goulet-Pelletier [61] method in the TOSTER package[63]. We also compared this to alternatives in both the effect size and variance computation for within-subject studies, which made little difference (eFigure 1). If a within-subject study only provided enough data for calculating between-subject Cohen's *d* and raw data was unavailable, we classified it as a between-subject study.

Some within-subject studies applied mixed-effects models (due to uneven subjects in each condition) or non-parametric methods to analyze the data. Since calculating Cohen's d_{rm} is not straightforward in these cases, we opted to treat these as between-subject studies if the sample size in each group was very low for a within-subject comparison or transform the raw data, respectively.

In total, out of the original within-subject studies included, 13 studies in the dopamine domain and 4 studies in the serotonin domain were treated as between-subject studies. For meta-analyses where we additionally analyzed subcomponents based on only within-subject studies, we computed Cohen's d_z. These included 3 additional serotonin and 5 additional dopamine studies.

1.4.1 Conversion of outcome measures into effect sizes and associated variances

For between-subject studies and studies treated as between-subject studies, the following equations were used:

1.4.1.1 Cohen's d from Means and Standard Deviations of 2 samples

$$d = \frac{M_1 - M_2}{SD_{pooled}}, \quad (1)$$

where *d* is Cohen's *d*, M_1 is the mean of one sample, M_2 is the mean of the other sample, SD_{pooled} is the pooled standard deviation of the two samples.

1.4.1.2 Pooled standard deviation of 2 samples

$$SD_{pooled} = \sqrt{\frac{(N_1 - 1)SD_1^2 + (N_2 - 1)SD_2^2}{N_1 + N_2 - 2}}, \quad (2)$$

where N_1 is the size of one sample, N_2 is the size of the other sample, SD_1 is the standard deviation of one sample, SD_2 is the standard deviation of the other sample.

1.4.1.3 Cohen's d from t-statistic

$$d = \frac{t}{\sqrt{\frac{1}{N_1} + \frac{1}{N_2}}}, \quad (3)$$

where *t* is the independent t-statistic reported.

1.4.1.4 Cohen's d from F-statistic

$$SD_{pooled} = \sqrt{\frac{F}{\frac{1}{N_1} + \frac{1}{2}}}, \quad (4)$$

where *F* is the F-statistic reported.

1.4.1.5 Variance on Cohen's d

$$var(d) = \frac{N_1 + N_2}{N_1 \times N_2} + \frac{d^2}{2(N_1 + N_2)} \quad (5)$$

For within-subject studies, Cohen's d_{rm} was computed, taking into account the correlation between measures, using the Cousineau and Goulet-Pelletier [61] method. This is useful for when effect sizes are being compared for studies that involve between and within subjects designs[60]. The TOSTER package in R was used, which uses the following equations[63]:

1.4.1.6 *Cohen's d_{rm} from Means, Standard Deviations and Correlation Coefficient of paired samples*

$$d_{rm} = \frac{M_1 - M_2}{S_{diff}} \times \sqrt{\frac{2}{(1-r)}} \tag{6}$$

where d_{rm} is Cohen's d_{rm}, M_1 is the mean of one condition (drug), M_2 is the mean of the other condition (placebo), S_{diff} is the standard deviation of the difference scores, and r is the correlation between the drug and placebo conditions.

1.4.1.7 *The standard deviation of the difference scores*

$$S_{diff} = \sqrt{SD_1^2 + SD_2^2 - 2 \times r \times SD_1 \times SD_2}, \tag{7}$$

where S_{diff} is the standard deviation of the difference scores of the two samples, SD_1 is the standard deviation of one condition, and SD_2 is the standard deviation of the other condition.

1.4.1.8 *The degrees of freedom for Cohen's d_{rm} under the Cousineau and Goulet-Pelletier (2021) method*

$$df = 2(N-1), \tag{8}$$

where N represents the sample size.

1.4.1.9 *The variance of Cohen's d_{rm}*

$$var(d_{rm}) = \frac{df}{df-2} \times \frac{2(1-r)}{N} \times (1 + d_{rm}^2 \times \frac{N}{2(1-r)}) - \frac{d_{rm}^2}{1} \tag{9}$$

For within-subject studies where we had M_1, M_2, SD_1, SD_2 and the paired t-statistic, we estimated the correlation coefficient r between conditions with the following procedure.

Compute standard error of the difference, *SED*:

$$SED = \left|\frac{M_1 - M_2}{t}\right| \tag{10}$$

Compute the standard deviation of the difference scores, S_{diff}:

$$S_{diff} = SED \times \sqrt{N} \tag{11}$$

Calculate covariance, *Cov*:

$$Cov = \frac{SD_1^2 + SD_2^2 - S_{diff}^2}{2} \tag{12}$$

The correlation coefficient *r* is:

$$r = \frac{Cov}{SD_1 \times SD_2} \tag{13}$$

For supplemental meta-analyses where we only included within-subject studies, we first computed a paired t-test if one was not already provided in the study and then computed Cohen's d_z with the following equation:

1.4.1.10 Cohen's d_z based on the t-statistic, t

$$d_z = \frac{t}{\sqrt{N}} \tag{14}$$

1.4.1.11 The variance of Cohen's d_z

$$var(d_z) = \frac{1}{N} + \frac{d_z^2}{2 \times N} \tag{15}$$

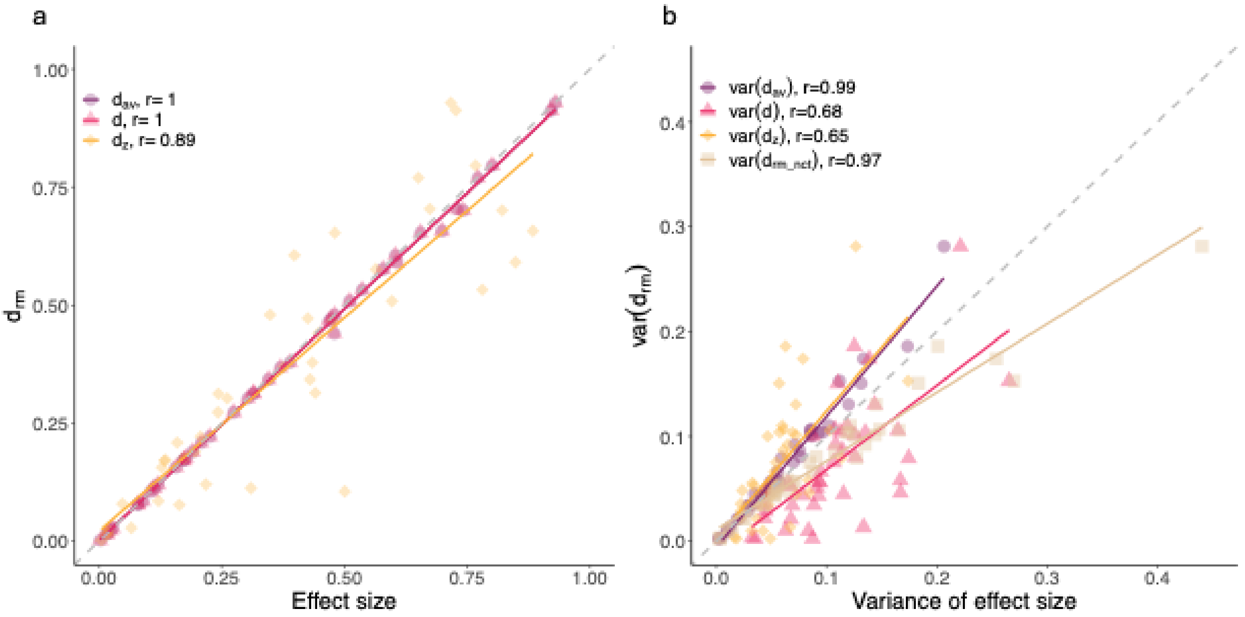

eFigure 1: Comparison of a) effect size and b) variance of effect size calculations for within-subject studies that provided sufficient data to compute all variations (N=42). The y-axis represents Cohen's d_{rm} and the x-axis represents Cohen's d_{av}, d, or d_z in figure a. $d_{av} = \frac{M_1 - M_2}{\sqrt{\frac{SD_1^2+SD_2^2}{2}}}$[59], and will equal d when the condition sample sizes are equal. In figure b, the y-axis represents the variance of Cohen's d_{rm} (equation 9) and the x-axis represents four other calculations of the variance. *Var(d)* represents the variance of Cohen's d (equation 5); *var(d_z)* represents the variance of d_z (equation 15); and *var(d_{av})* represents the variance of d_{av}. *Var(d_{av})* is calculated as: $var(d_{av}) = (\frac{CIup - CIlow}{2\times 1.96})^2$, where CI is calculated using the non-central *t* distribution: $CI = d_{av} \pm t(1-\frac{\alpha}{2}, n-1, \lambda) \times \sqrt{\frac{2(SD_1^2+SD_2^2-2\times r\times SD_1\times SD_2)}{N(SD_1^2+SD_2^2)}}$, α=0.05 and the non-centrality parameter is the observed t-value: $\lambda = d_{av} \times \sqrt{\frac{N(SD_1^2+SD_2^2)}{2(SD_1^2+SD_2^2-2\times r\times SD_1\times SD_2)}}$[64]. *Var($d_{rm\_nct}$)* represents the variance of d_{rm} calculated with the setting "nct" for the confidence interval type in the package TOSTER[63], which uses the same equation as in equation 9 but with $df = N-1$. The gray dished diagonal line represents the reference line when y=x.

2 Supplementary results

	Dopamine			Serotonin		
**Category**	**No. of studies**	**Placebo N**	**Drug N**	**No. of studies**	**Placebo N**	**Drug N**
Overall reward	49	1260	1266	21	572	559
Overall punishment	17	362	365	11	292	285
Reward learning/sensitivity	25	616	623	9	274	269
Reward discounting	12	439	436	4	74	74
Reward response vigour	9	185	186	4	96	94
Appetitive Pavlovian	5	158	157	4	128	122
Punishment learning/sensitivity	13	244	249	4	108	106
Aversive Pavlovian	4	118	116	7	184	179
Model-based learning/flexibility	8	236	239	10	306	300
Risk attitude	5	155	152	6	105	104
Aversive Pavlovian within-subjects	-	-	-	6	145	-
Risk attitude within-subjects	5	127	-	-	-	-
Model-based learning/flexibility within-subjects	8	198	-	-	-	-

eTable 2: Number of studies and participants (N) for each condition for each assessed category.

2.1 Assessment of study quality

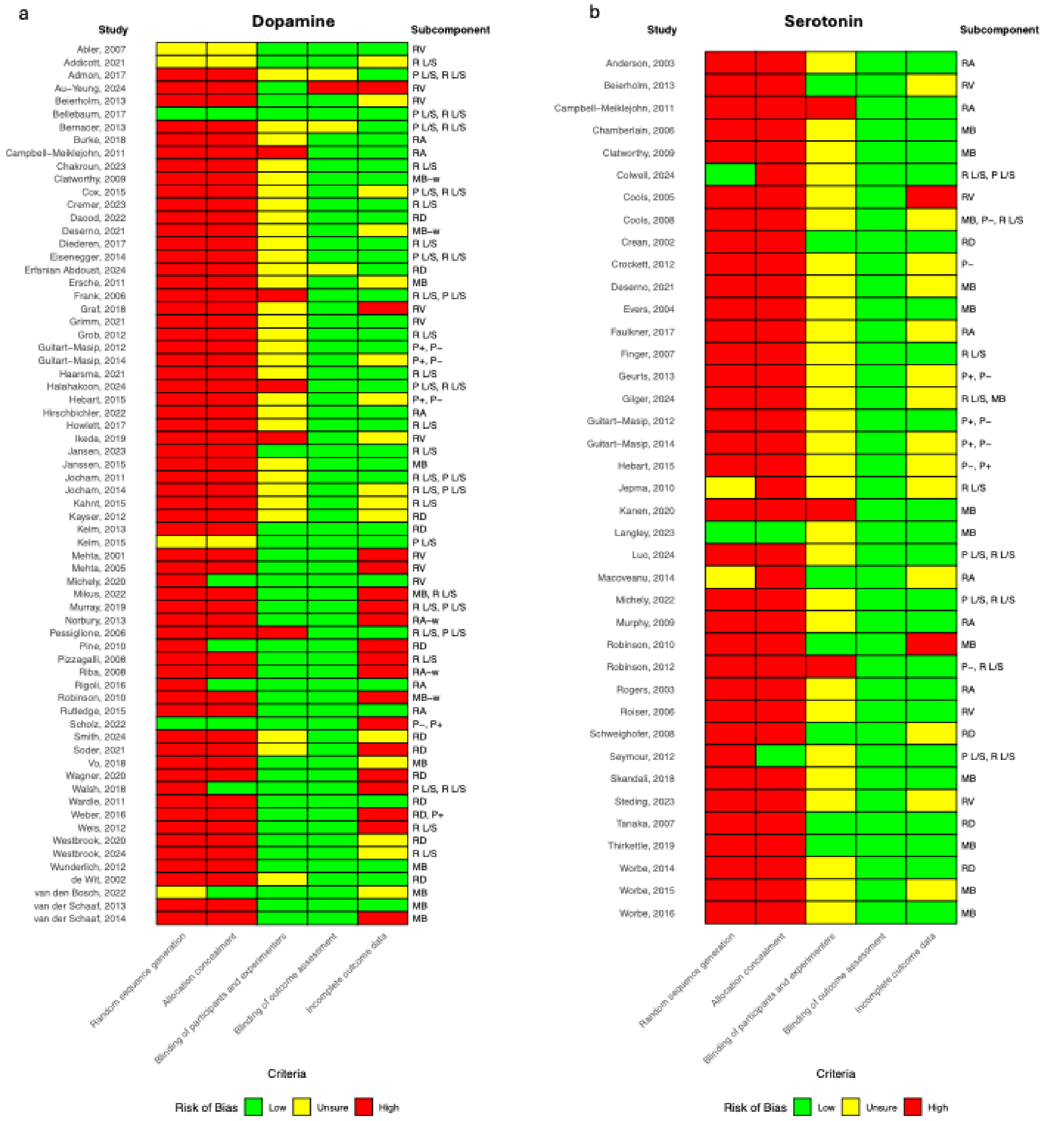

eFigure 2: Risk of bias assessment for a) dopamine and b) serotonin studies. Abbreviations: R L/S: reward learning sensitivity; P L/S: punishment learning/sensitivity; P+: appetitive Pavlovian; P-: aversive Pavlovian; RD: reward discounting; RV: reward vigor; MB: model-based learning/flexibility; RA: risk attitude: -w: within-subjects study only

2.2 Additional dopamine results

a

Study	N(Drug, Pla)	Drug, dose	SMD [95% CI]
Jocham, 2011	16	Amisulpride 200mg	0.93 [0.11, 1.75] *
Admon, 2017	20, 18	Amisulpride 50mg	0.26 [−0.38, 0.90] *
Frank, 2006	19, 20	Haloperidol 2mg	0.37 [−0.26, 1.00] *
Wagner, 2020	23, 26	Haloperidol 2mg	0.53 [−0.04, 1.10] *
Erfanian Abdoust, 2024	62	Haloperidol 2mg	0.00 [−0.12, 0.12] *
Walsh, 2018	20, 20	Bupropion 150mg	−0.06 [−0.68, 0.56]
Ikeda, 2019	15	Bupropion 150mg	0.30 [−0.33, 0.93]
de Wit, 2002	36, 36	D−amphetamine 20mg	0.21 [−0.25, 0.67]
Wardle, 2011	17, 17	D−amphetamine 20mg	0.64 [−0.05, 1.33]
Soder, 2021	28, 28	D−amphetamine 20mg	0.33 [−0.20, 0.86]
Bernacer, 2013	17	Methamphetamine 0.3mg/kg	−0.70 [−1.33, −0.08]
Westbrook, 2020	46	Methylphenidate 20mg	0.19 [−0.10, 0.48]
Daood, 2022	57	Methylphenidate 20mg	0.11 [0.01, 0.21]
Howlett, 2017	19	Methylphenidate 40mg	0.27 [−0.26, 0.81]
Addicott, 2021	22	Methylphenidate 40mg	0.38 [−0.03, 0.79]
Bellebaum, 2017	16, 20	Modafinil 200mg	0.38 [−0.29, 1.04]
Pessiglione, 2006	13, 13	L−dopa 100mg+25mg	0.75 [−0.04, 1.55]
Grimm, 2021	45	L−dopa 100mg+25mg	0.02 [−0.35, 0.39]
Jansen, 2023	30, 30	L−dopa 100mg+25mg	0.00 [−0.51, 0.51]
Weis, 2012	27, 28	L−dopa 100mg+35mg	0.04 [−0.49, 0.57]
Pine, 2010	13	L−dopa 150mg	−0.70 [−1.34, −0.06]
Michely, 2020	20	L−dopa 150mg	0.80 [0.20, 1.39]
Smith, 2024	76, 76	L−dopa 150mg	0.14 [−0.18, 0.46]
Guitart−Masip, 2012	16, 20	L−dopa 150mg+37.5mg	−0.10 [−0.76, 0.56]
Beierholm, 2013	30, 30	L−dopa 150mg+37.5mg	0.59 [0.07, 1.11]
Guitart−Masip, 2014	30, 29	L−dopa 150mg+37.5mg	−0.30 [−0.82, 0.21]
Chakroun, 2023	31	L−dopa 150mg+37.5mg	−0.17 [−0.63, 0.29]
Mehta, 2001	18	Bromocriptine 1.25mg	0.47 [−0.10, 1.05]
Au−Yeung, 2024	19, 18	Pramipexole 1mg/day	0.21 [−0.44, 0.86]
Halahakoon, 2024	21, 19	Pramipexole 1mg/day	0.68 [0.04, 1.32]
Kayser, 2012	23	Tolcapone 200mg	0.11 [0.01, 0.20]
Scholz, 2022	35, 35	Tolcapone 200mg	0.03 [−0.44, 0.50]
Graf, 2018	17	Amisulpride 200mg/day	0.21 [−0.44, 0.86]
Jocham, 2014	22	Amisulpride 400mg	0.44 [−0.02, 0.90]
Kahnt, 2015	27, 24	Amisulpride 400mg	0.24 [−0.31, 0.79]
Weber, 2016	40, 40	Amisulpride 400mg	−0.58 [−1.02, −0.13]
Murray, 2019	18, 18	Amisulpride 400mg	−0.04 [−0.69, 0.62]
Mikus, 2022	38, 35	Amisulpride 400mg	0.38 [−0.08, 0.84]
Cremer, 2023	23, 22	Amisulpride 400mg	0.59 [−0.00, 1.19]
Abler, 2007	8	Olanzapine 5mg	0.66 [−0.11, 1.42]
Diederen, 2017	19, 19	Sulpiride 600mg	0.58 [−0.07, 1.23]
Haarsma, 2021	20, 20	Sulpiride 600mg	0.71 [0.07, 1.35]
Eisenegger, 2014	41, 35	Sulpiride 800mg	−0.05 [−0.50, 0.40]
Grob, 2012	28, 28	AMPT 40mg/kg	−0.36 [−0.88, 0.17]
Cox, 2015	10, 10	Phe / Tyr depletion 100g	0.00 [−0.88, 0.88]
Mehta, 2005	14	Phe / Tyr depletion	−0.17 [−0.88, 0.54]
Hebart, 2015	35, 34	Phe / Tyr depletion	0.65 [0.17, 1.14]
Kelm, 2013	15	Phe / Tyr depletion 68.7g	−0.03 [−0.26, 0.20]
Pizzagalli, 2008	11, 13	Pramipexole 0.5mg	1.42 [0.52, 2.32] *
Random−effects meta−analysis:			0.18 [0.09, 0.28]

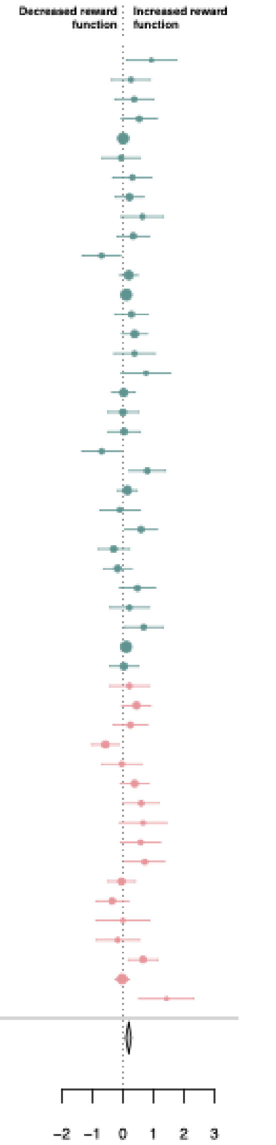

b

Study	N(Drug, Pla)	Drug, dose	SMD [95% CI]
Jocham, 2011	16	Amisulpride 200mg	0.08 [−0.77, 0.92]*
Admon, 2017	20, 18	Amisulpride 50mg	0.54 [−0.11, 1.19]*
Frank, 2006	19, 20	Haloperidol 2mg	−0.38 [−1.01, 0.25]*
Walsh, 2018	20, 20	Bupropion 150mg	0.37 [−0.25, 1.00]
Bernacer, 2013	17, 17	Methamphetamine 0.3mg/kg	−0.01 [−0.68, 0.66]
Bellebaum, 2017	16, 20	Modafinil 200mg	−0.52 [−1.19, 0.14]
Pessiglione, 2006	13, 13	L−dopa 100mg+25mg	0.42 [−0.35, 1.20]
Guitart−Masip, 2012	16, 20	L−dopa 150mg+37.5mg	0.19 [−0.47, 0.85]
Guitart−Masip, 2014	30, 29	L−dopa 150mg+37.5mg	−0.21 [−0.72, 0.30]
Halahakoon, 2024	21, 19	Pramipexole 1mg/day	−0.63 [−1.27, 0.00]
Scholz, 2022	35, 35	Tolcapone 200mg	−0.09 [−0.56, 0.37]
Jocham, 2014	22	Amisulpride 400mg	0.58 [0.07, 1.08]
Murray, 2019	18, 18	Amisulpride 400mg	−0.51 [−1.17, 0.16]
Eisenegger, 2014	41, 35	Sulpiride 800mg	0.22 [−0.23, 0.67]
Cox, 2015	10	Phe / Tyr depletion 100g	−0.91 [−1.95, 0.13]
Hebart, 2015	35, 34	Phe / Tyr depletion	−0.40 [−0.88, 0.07]
Kelm, 2015	16	Phe / Tyr depletion 68.7g	−0.22 [−0.78, 0.33]
Random−effects meta−analysis:			−0.06 [−0.26, 0.13]

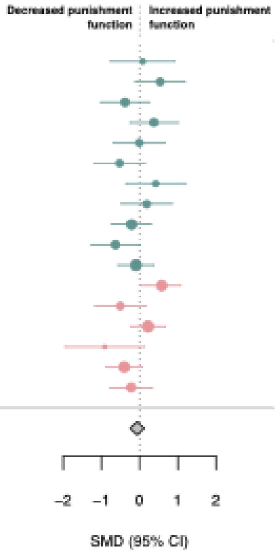

eFigure 3: The effect of upregulating dopamine on overall reward and punishment. Standardized mean differences (SMDs) between the effect of upregulating dopamine versus placebo on a) overall reward function, and b) overall punishment function. Pla indicates placebo. We recoded antagonist effects as if they were agonists (pink SMD point estimates and 95% CI). Green SMD point estimates and 95% CI indicate studies that were coded as original agonists. Asterisks indicate studies that used a low dose of an agonist or antagonists. These effects were interpreted contrary to their typical activity profile (e.g., a low-dose agonist acting antagonistically), as suggested by the literature.

eFigure 4: The effect of upregulating dopamine on overall reward and punishment according to original drug profile. Standardized mean differences (SMDs) between the effect of upregulating dopamine versus placebo on a) overall reward function, and b) overall punishment function according to the original drug activity profile. Pla indicates placebo. We recoded antagonist effects as if they were agonists (pink SMD point estimates and 95% CI). Green SMD point estimates and 95% CI indicate studies that were coded as original agonists.

a

Study	N(Drug, Pla)	Drug, dose	SMD [95% CI]
Walsh, 2018	20, 20	Bupropion 150mg	−0.06 [−0.68, 0.56]
Ikeda, 2019	15	Bupropion 150mg	0.30 [−0.33, 0.93]
de Wit, 2002	36, 36	D−amphetamine 20mg	0.21 [−0.25, 0.67]
Wardle, 2011	17, 17	D−amphetamine 20mg	0.64 [−0.05, 1.33]
Soder, 2021	28, 28	D−amphetamine 20mg	0.33 [−0.20, 0.86]
Bernacer, 2013	17	Methamphetamine 0.3mg/kg	−0.70 [−1.33, −0.08]
Westbrook, 2020	46	Methylphenidate 20mg	0.19 [−0.10, 0.48]
Daood, 2022	57	Methylphenidate 20mg	0.11 [0.01, 0.21]
Howlett, 2017	19	Methylphenidate 40mg	0.27 [−0.26, 0.81]
Addicott, 2021	22	Methylphenidate 40mg	0.38 [−0.03, 0.79]
Bellebaum, 2017	16, 20	Modafinil 200mg	0.38 [−0.29, 1.04]
Pessiglione, 2006	13, 13	L−dopa 100mg+25mg	0.75 [−0.04, 1.55]
Grimm, 2021	45	L−dopa 100mg+25mg	0.02 [−0.35, 0.39]
Jansen, 2023	30, 30	L−dopa 100mg+25mg	0.00 [−0.51, 0.51]
Weis, 2012	27, 28	L−dopa 100mg+35mg	0.04 [−0.49, 0.57]
Pine, 2010	13	L−dopa 150mg	−0.70 [−1.34, −0.06]
Michely, 2020	20	L−dopa 150mg	0.80 [0.20, 1.39]
Smith, 2024	76, 76	L−dopa 150mg	0.14 [−0.18, 0.46]
Guitart−Masip, 2012	16, 20	L−dopa 150mg+37.5mg	−0.10 [−0.76, 0.56]
Beierholm, 2013	30, 30	L−dopa 150mg+37.5mg	0.59 [0.07, 1.11]
Guitart−Masip, 2014	30, 29	L−dopa 150mg+37.5mg	−0.30 [−0.82, 0.21]
Chakroun, 2023	31	L−dopa 150mg+37.5mg	−0.17 [−0.63, 0.29]
Mehta, 2001	18	Bromocriptine 1.25mg	0.47 [−0.10, 1.05]
Pizzagalli, 2008	11, 13	Pramipexole 0.5mg	−1.42 [−2.32, −0.52]
Au−Yeung, 2024	19, 18	Pramipexole 1mg/day	0.21 [−0.44, 0.86]
Halahakoon, 2024	21, 19	Pramipexole 1mg/day	0.68 [0.04, 1.32]
Kayser, 2012	23	Tolcapone 200mg	0.11 [0.01, 0.20]
Scholz, 2022	35, 35	Tolcapone 200mg	0.03 [−0.44, 0.50]
Jocham, 2011	16	Amisulpride 200mg	−0.93 [−1.75, −0.11]
Graf, 2018	17	Amisulpride 200mg/day	0.21 [−0.44, 0.86]
Jocham, 2014	22	Amisulpride 400mg	0.44 [−0.02, 0.90]
Kahnt, 2015	27, 24	Amisulpride 400mg	0.24 [−0.31, 0.79]
Weber, 2016	40, 40	Amisulpride 400mg	−0.58 [−1.02, −0.13]
Murray, 2019	18, 18	Amisulpride 400mg	−0.04 [−0.69, 0.62]
Mikus, 2022	38, 35	Amisulpride 400mg	0.38 [−0.08, 0.84]
Cremer, 2023	23, 22	Amisulpride 400mg	0.59 [−0.00, 1.19]
Admon, 2017	20, 18	Amisulpride 50mg	−0.26 [−0.90, 0.38]
Frank, 2006	19, 20	Haloperidol 2mg	−0.37 [−1.00, 0.26]
Wagner, 2020	23, 26	Haloperidol 2mg	−0.53 [−1.10, 0.04]
Erfanian Abdoust, 2024	62	Haloperidol 2mg	−0.00 [−0.12, 0.12]
Abler, 2007	8	Olanzapine 5mg	0.66 [−0.11, 1.42]
Diederen, 2017	19, 19	Sulpiride 600mg	0.58 [−0.07, 1.23]
Haarsma, 2021	20, 20	Sulpiride 600mg	0.71 [0.07, 1.35]
Eisenegger, 2014	41, 35	Sulpiride 800mg	−0.05 [−0.50, 0.40]
Grob, 2012	28, 28	AMPT 40mg/kg	−0.36 [−0.88, 0.17]
Cox, 2015	10, 10	Phe / Tyr depletion 100g	0.00 [−0.88, 0.88]
Mehta, 2005	14	Phe / Tyr depletion	−0.17 [−0.88, 0.54]
Hebart, 2015	35, 34	Phe / Tyr depletion	0.65 [0.17, 1.14]
Kelm, 2013	15	Phe / Tyr depletion 68.7g	−0.03 [−0.26, 0.20]
Random−effects meta−analysis:			0.10 [−0.00, 0.20]

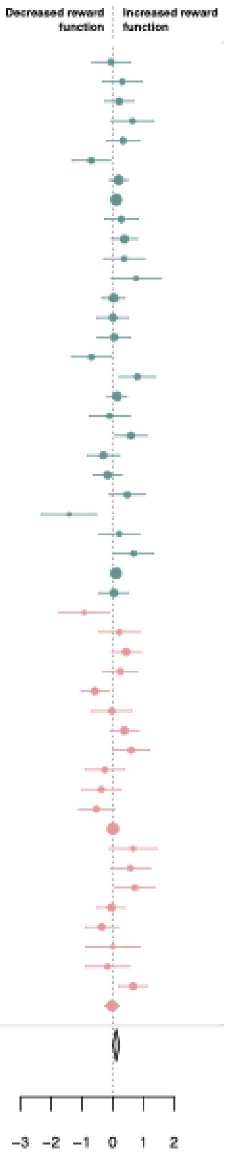

b

Study	N(Drug, Pla)	Drug, dose	SMD [95% CI]
Walsh, 2018	20, 20	Bupropion 150mg	0.37 [−0.25, 1.00]
Bernacer, 2013	17, 17	Methamphetamine 0.3mg/kg	−0.01 [−0.68, 0.66]
Bellebaum, 2017	16, 20	Modafinil 200mg	−0.52 [−1.19, 0.14]
Pessiglione, 2006	13, 13	L−dopa 100mg+25mg	0.42 [−0.35, 1.20]
Guitart−Masip, 2012	16, 20	L−dopa 150mg+37.5mg	0.19 [−0.47, 0.85]
Guitart−Masip, 2014	30, 29	L−dopa 150mg+37.5mg	−0.21 [−0.72, 0.30]
Halahakoon, 2024	21, 19	Pramipexole 1mg/day	−0.63 [−1.27, 0.00]
Scholz, 2022	35, 35	Tolcapone 200mg	−0.09 [−0.56, 0.37]
Jocham, 2011	16	Amisulpride 200mg	−0.08 [−0.92, 0.77]
Jocham, 2014	22	Amisulpride 400mg	0.58 [0.07, 1.08]
Murray, 2019	18, 18	Amisulpride 400mg	−0.51 [−1.17, 0.16]
Admon, 2017	20, 18	Amisulpride 50mg	−0.54 [−1.19, 0.11]
Frank, 2006	19, 20	Haloperidol 2mg	0.38 [−0.25, 1.01]
Eisenegger, 2014	41, 35	Sulpiride 800mg	0.22 [−0.23, 0.67]
Cox, 2015	10	Phe / Tyr depletion 100g	−0.91 [−1.95, 0.13]
Hebart, 2015	35, 34	Phe / Tyr depletion	−0.40 [−0.88, 0.07]
Kelm, 2015	16	Phe / Tyr depletion 68.7g	−0.22 [−0.78, 0.33]
Random−effects meta−analysis:			−0.09 [−0.28, 0.11]

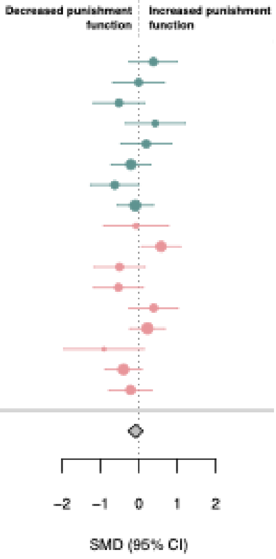

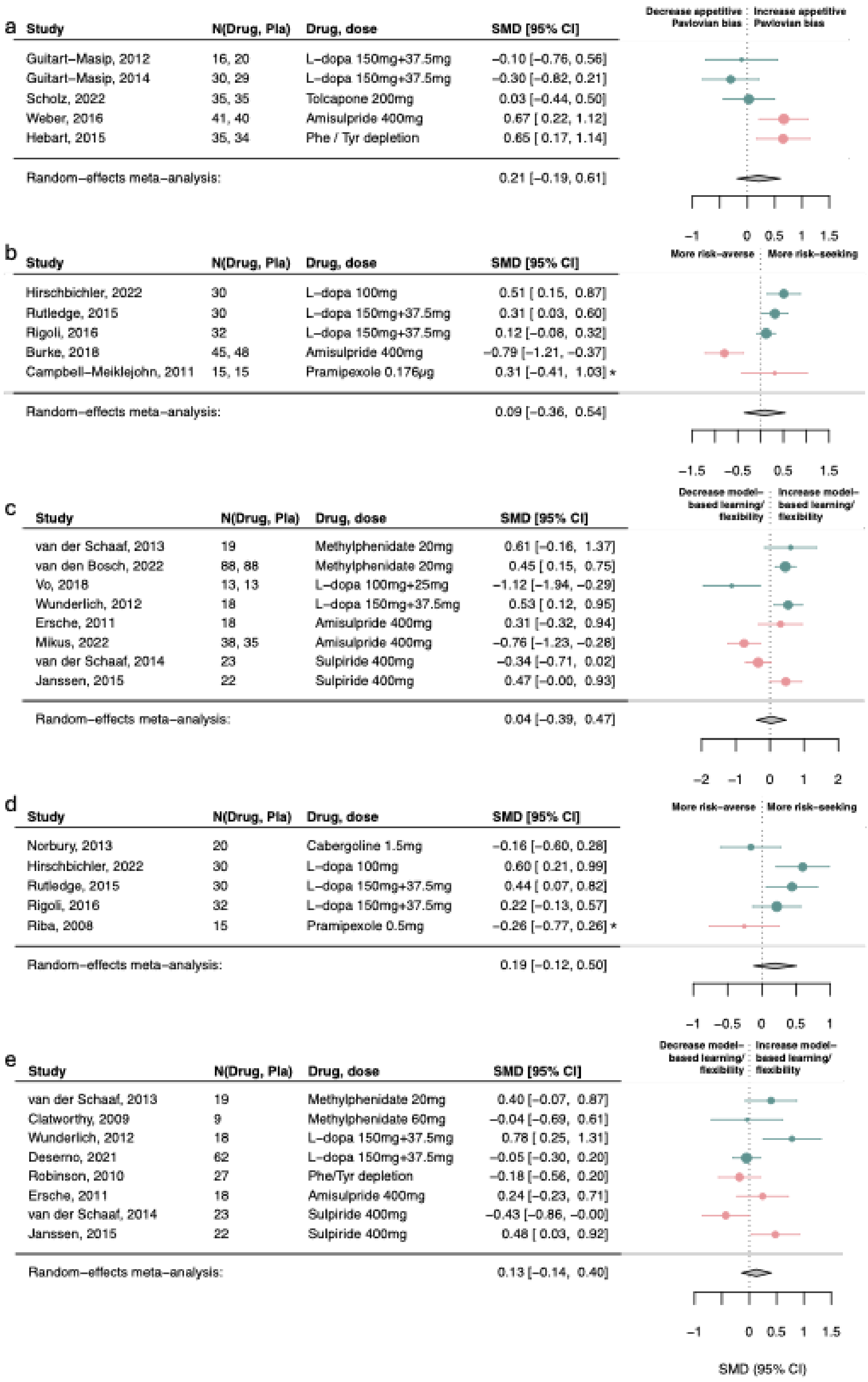

eFigure 5: The effect of upregulating dopamine on appetitive Pavlovian bias, risk and model-based learning/flexibility. Standardized mean differences (SMDs) between the effect of upregulating dopamine versus placebo on a) appetitive Pavlovian bias, b) risk attitude, c) model-based learning/flexibility, d) risk attitude in within-subject studies only, and e) model-based learning/flexibility in within-subjects only. Forest plots d and e display SMDs that reflect Cohen's d_z. Pla indicates placebo. We recoded antagonist effects as if they were agonists (pink SMD point estimates and 95% CI). Green SMD point estimates and 95% CI indicate studies that were coded as original agonists. Asterisks indicate studies that used a low dose of an agonist or antagonists. These effects were interpreted contrary to their typical activity profile (e.g., a low-dose agonist acting antagonistically), as suggested by the literature.

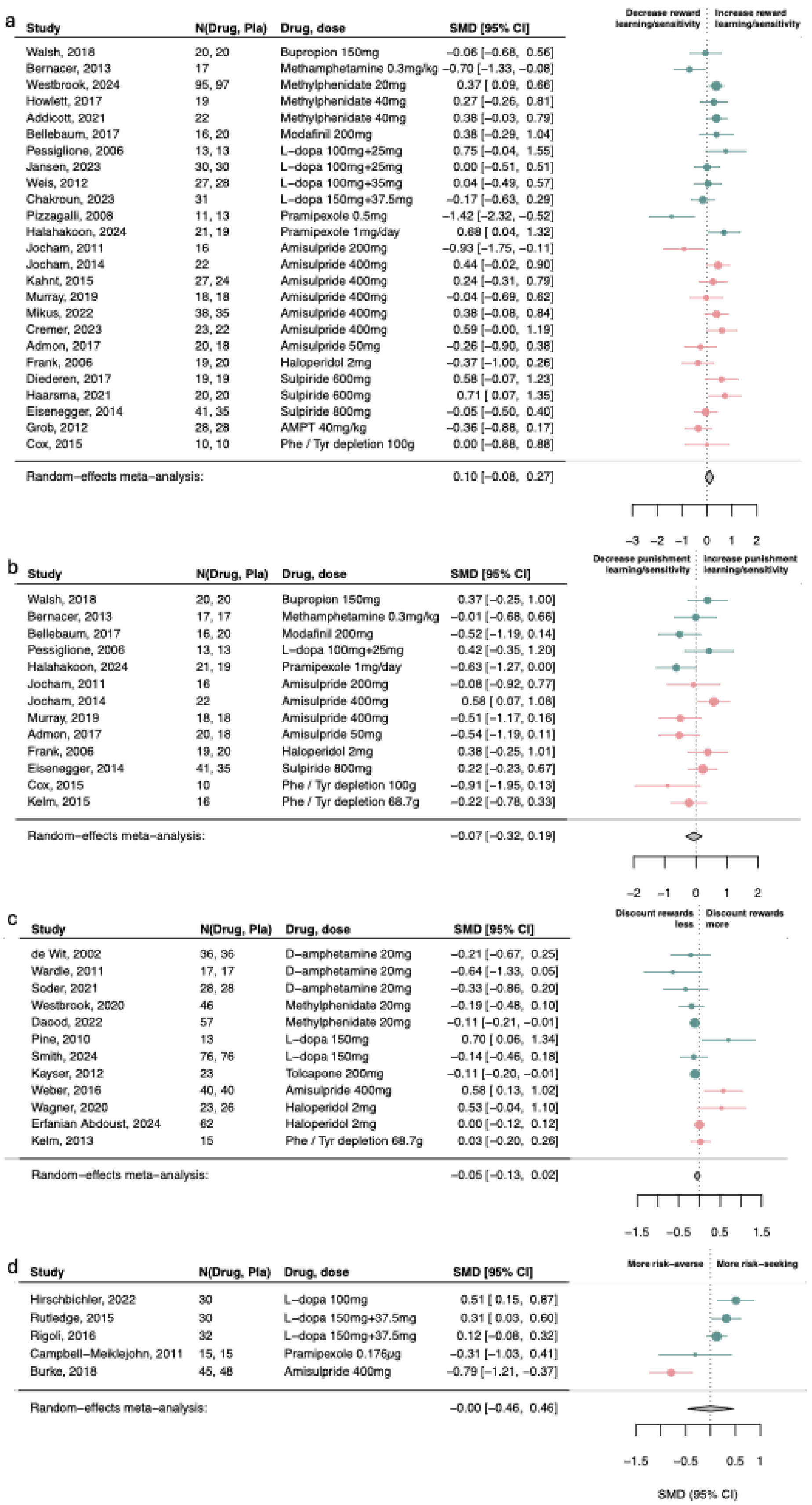

eFigure 6: The effect of upregulating dopamine on subcomponents according to original drug profile. The figure shows standardized mean differences (SMDs) between the effect of upregulating dopamine versus placebo on a) reward learning/sensitivity, b) punishment learning/sensitivity, c) reward discounting and d) risk attitude according to the original drug activity profile. Pla indicates placebo. We recoded antagonist effects as if they were agonists (pink SMD point estimates and 95% CI). Green SMD point estimates and 95% CI indicate studies that were coded as original agonists.

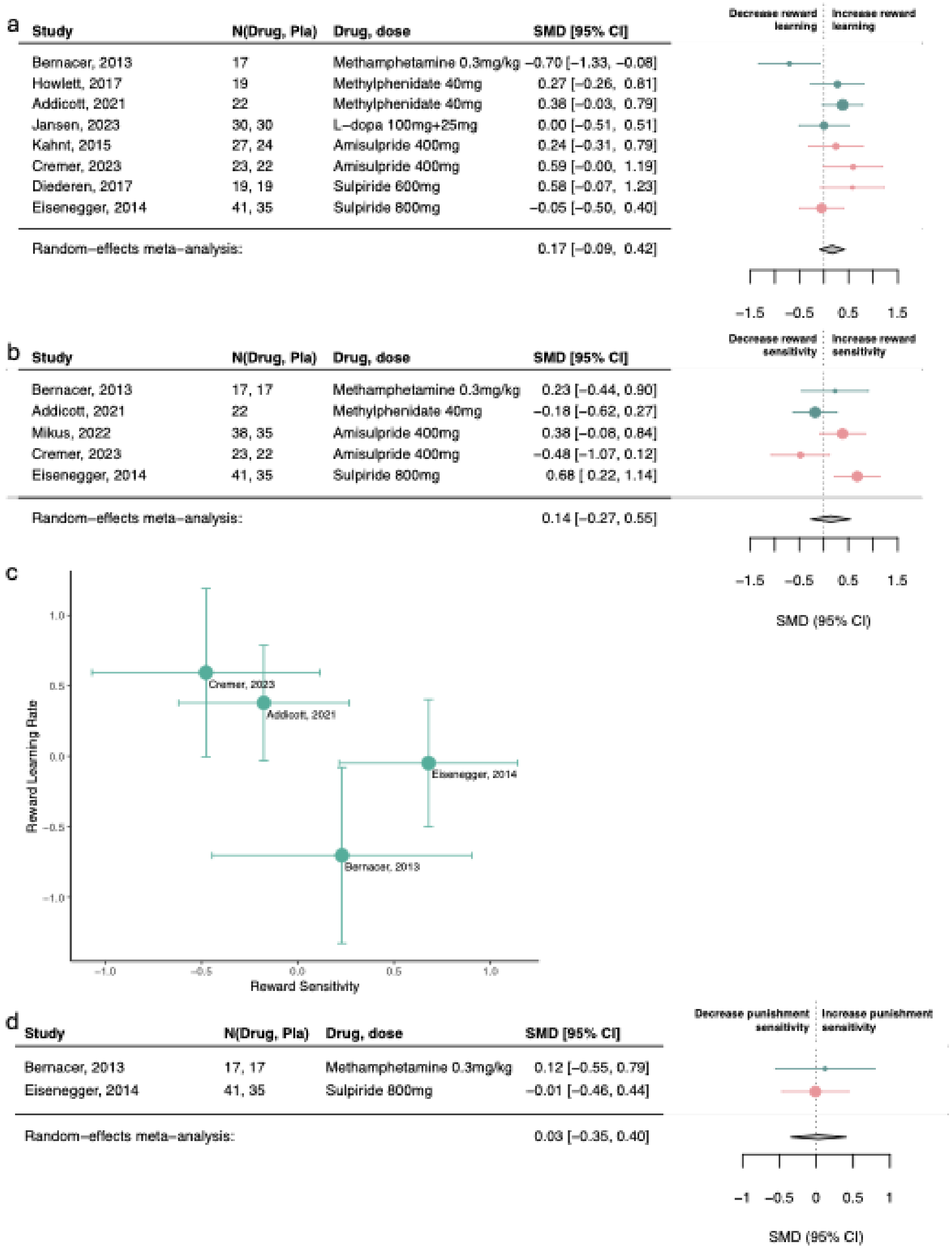

eFigure 7: The effect of upregulating dopamine on learning rate and sensitivity parameters. Standardized mean differences (SMDs) between the effect of upregulating dopamine versus placebo on a) reward learning rates, b) reward sensitivity, c) scatter plot of reward learning rate and reward sensitivity SMDs for studies that reported both - error bars represent 95% CI, and d) punishment sensitivity. Pla indicates placebo. We recoded antagonist effects as if they were agonists (pink SMD point estimates and 95% CI). Green SMD point estimates and 95% CI indicate studies that were coded as original agonists.

2.3 Dopamine heterogeneity

There was low-to-medium heterogeneity in overall reward (τ^2=0.04, I^2=58.50%) and overall punishment processes (τ^2=0.07, I^2=41.74%). Excluding four identified outlier studies[2,27,47,65] reduced heterogeneity to low in overall reward processes (τ^2=0.01; I^2=31.39%), but did not substantially affect the pooled effect size (SMD=0.19, 95% CI [0.11, 0.26]).

There was low heterogeneity in the reward learning/sensitivity subcomponent (τ^2=0.05, I^2=39.31%; removing two identified outliers[2,27] reduced this heterogeneity further τ^2=0.01, I^2=12.94%, which did not significantly affect the pooled effect size (SMD=0.26, 95% CI [0.14, 0.38]). There was moderate-to-substantial heterogeneity in the punishment learning/sensitivity (τ^2=0.11, I^2=50.74%), appetitive Pavlovian (τ^2=0.14, I^2=67.66%), risk attitude (τ^2=0.22, I^2=88.22%), and model-based learning/flexibility subcomponent (τ^2=0.31, I^2=84.76%). Removing an identified outlier in the risk attitude subcomponent[66] reduced heterogeneity to τ^2=0.01, I^2=32.98%, which resulted in a significant SMD estimate (SMD=0.27, 95% CI [0.08, 0.47]; eFigure 8). There was no-to-low heterogeneity in the reward response vigor (τ^2=0.02, I^2=19.18%), reward discounting (τ^2=0.0009, I^2=7.35%), and aversive Pavlovian (τ^2<0, I^2<0%) subcomponent. No other outliers were detected in either the subcategories or overall sample.

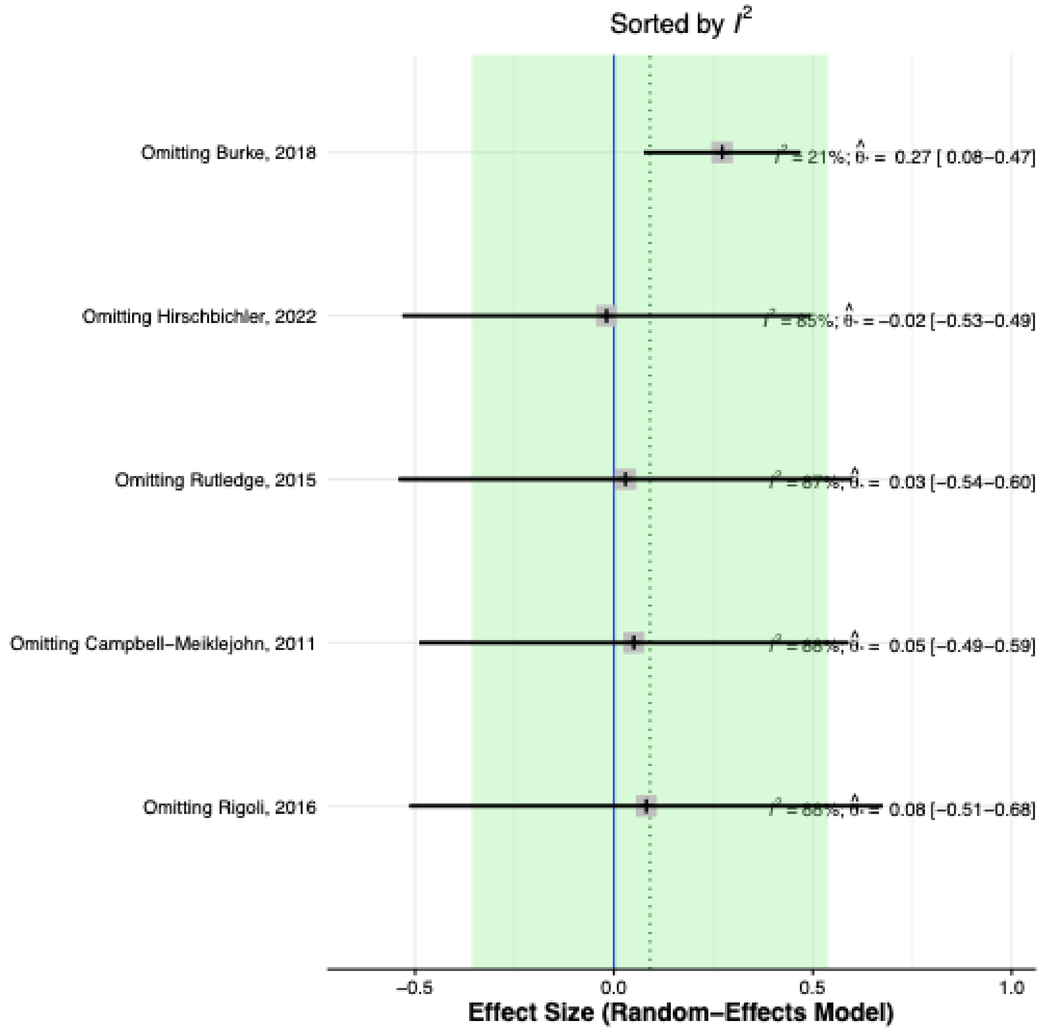

529 **eFigure 8: Leave-one-out meta-analysis for the effect of dopamine on risk attitude.** It is ordered by heterogeneity (low to high), displaying the recalculated pooled effect size and 95% confidence interval with one study omitted each time. The green shaded area represents the 95% confidence interval of the original pooled effect size, and the dotted line represents the original estimated pooled effect size.

2.4 Additional serotonin results

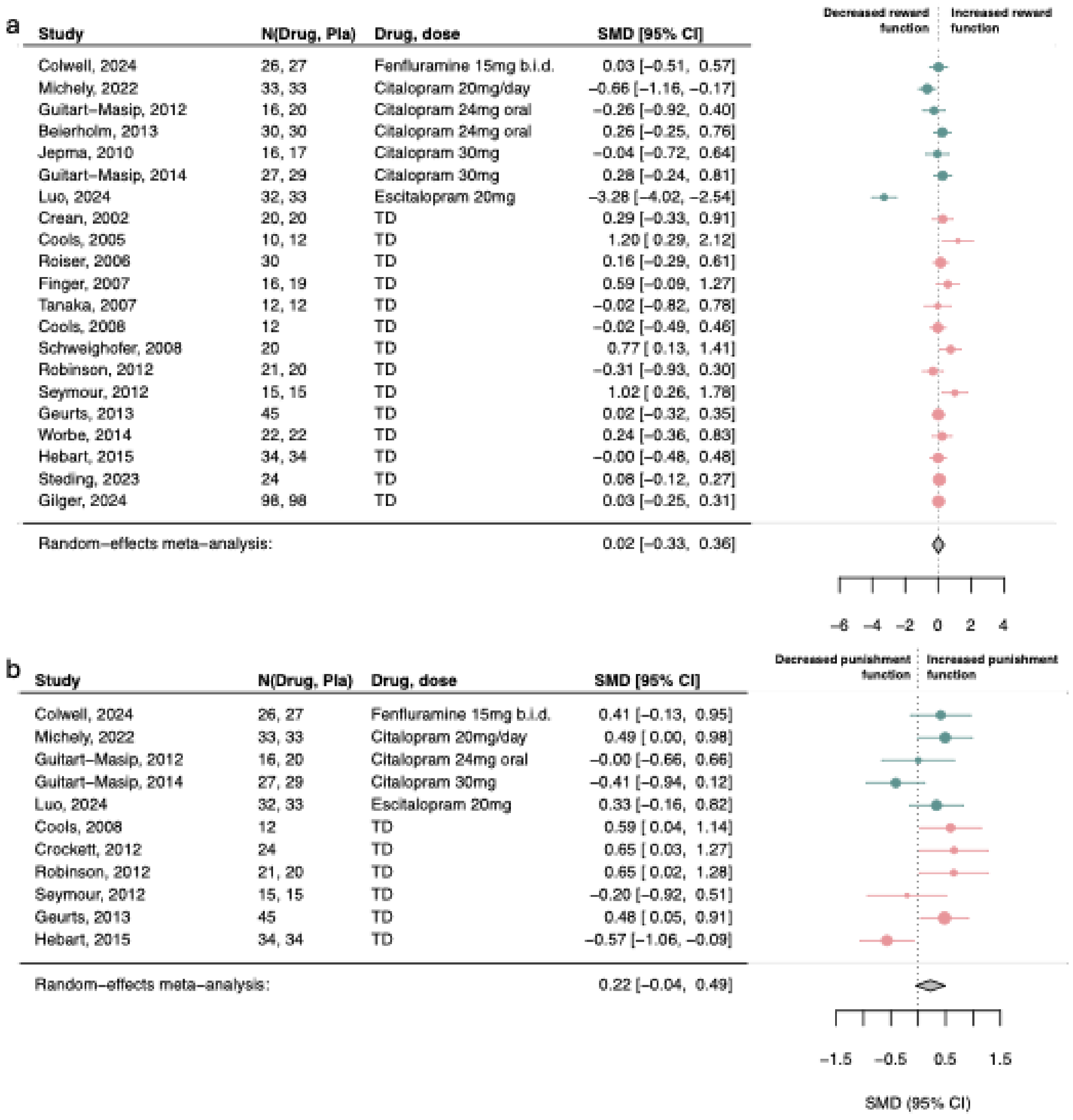

eFigure 9: The effect of upregulating serotonin on overall reward and punishment. Standardized mean differences (SMDs) between the effect of upregulating serotonin versus placebo on a) overall reward function, and b) overall punishment function. Pla indicates placebo. We recoded antagonist effects as if they were agonists (pink SMD point estimates and 95% CI). Green SMD point estimates and 95% CI indicate studies that were coded as original agonists. TD: tryptophan depletion.

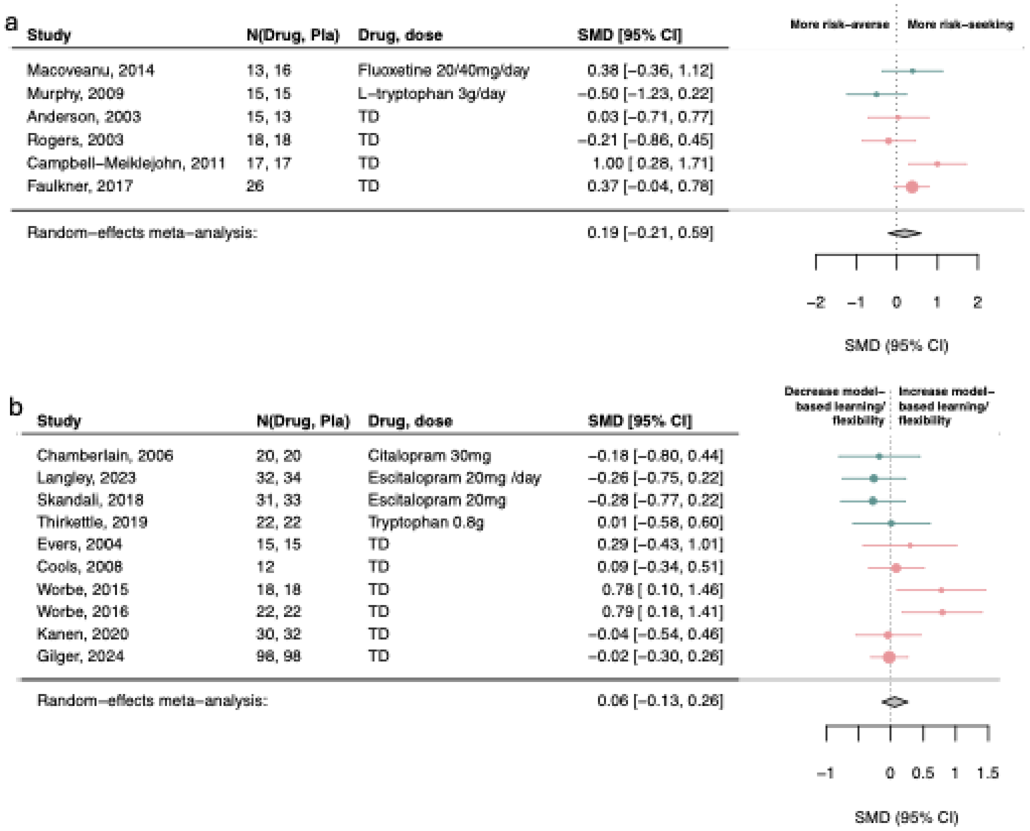

eFigure 10: The effect of upregulating serotonin on risk attitude and model-based learning/flexibility. Standardized mean differences (SMDs) between the effect of upregulating serotonin versus placebo on a) risk attitude, and b) model-based learning/flexibility. Pla indicates placebo. We recoded antagonist effects as if they were agonists (pink SMD point estimates and 95% CI). Green SMD point estimates and 95% CI indicate studies that were coded as original agonists. TD: tryptophan depletion.

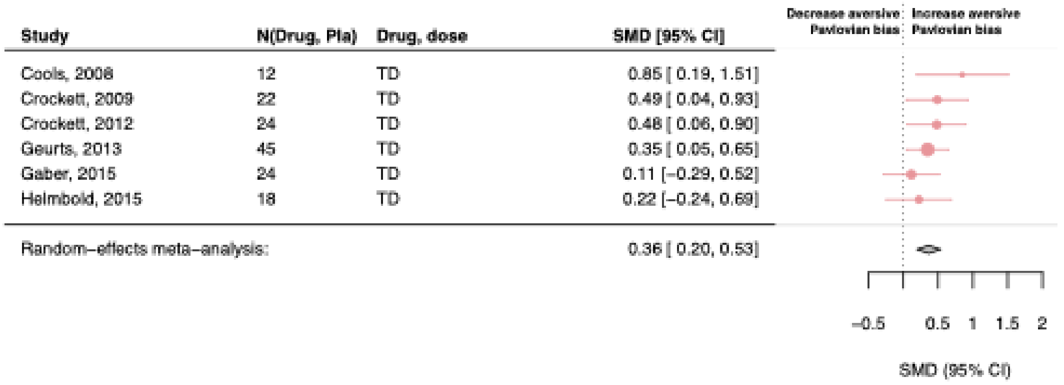

eFigure 11: The effect of upregulating serotonin on aversive Pavlovian biases in within-subject studies. Standardized mean differences (SMDs) between the effect of upregulating serotonin versus placebo on aversive Pavlovian bias, only including within-subject studies. These SMDs reflect Cohen's d_z. Pla indicates placebo. We recoded antagonist effects as if they were agonists (pink SMD point estimates and 95% CI). TD: tryptophan depletion.

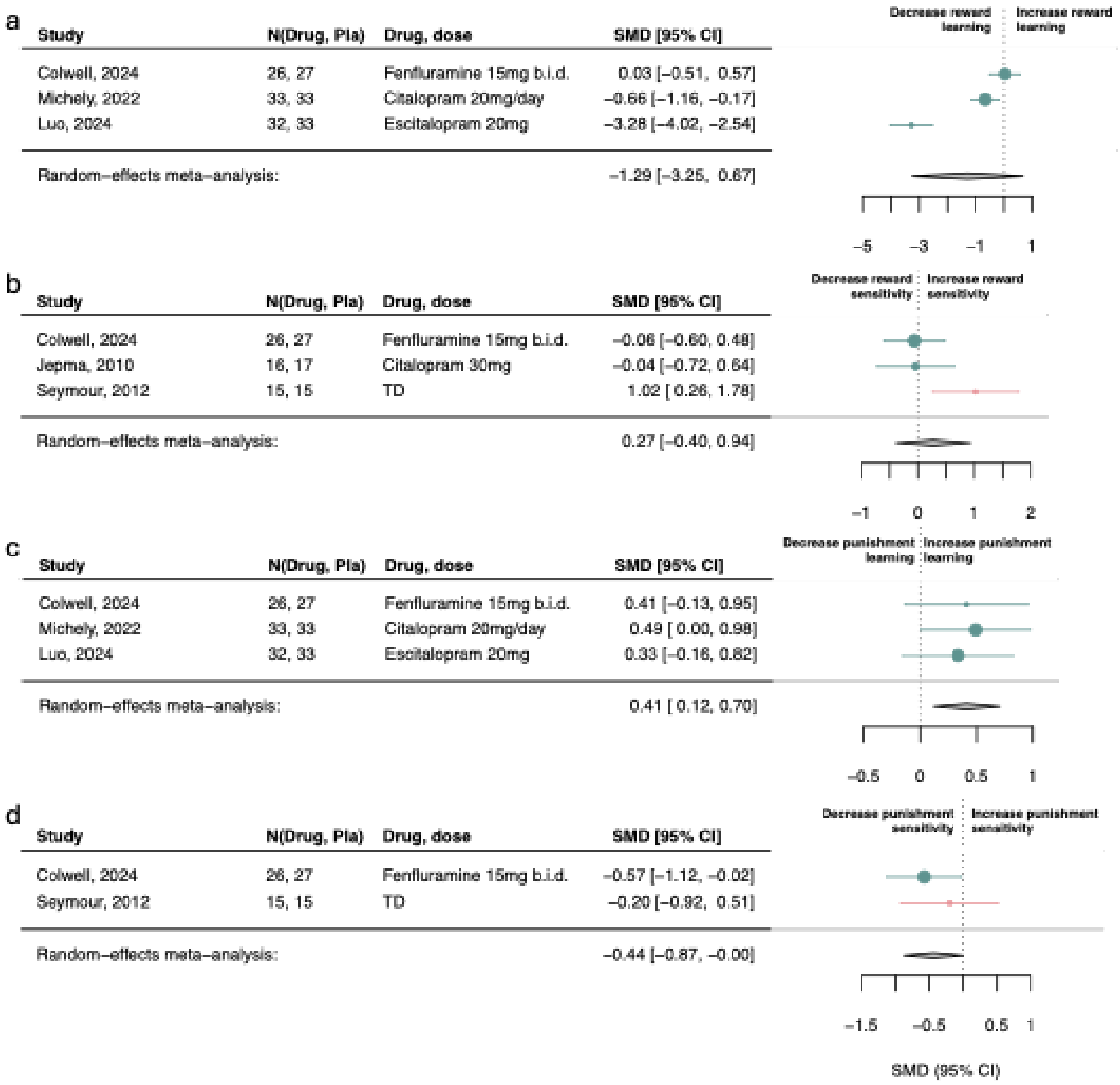

eFigure 12: The effect of upregulating serotonin on learning rate and sensitivity parameters. Standardized mean differences (SMDs) between the effect of upregulating serotonin versus placebo on a) reward learning rates, b) reward sensitivity, c) punishment learning rates, and d) punishment sensitivity. Pla indicates placebo. We recoded antagonist effects as if they were agonists (pink SMD point estimates and 95% CI). Green SMD point estimates and 95% CI indicate studies that were coded as original agonists. TD: tryptophan depletion.

2.5 Serotonin heterogeneity

Between-subject heterogeneity in overall reward (τ^2= 0.55, I^2= 90.44%) and punishment (τ^2= 0.12, I^2= 61.32%) processes were moderate-to-substantial. Removing one outlier study[67] in the overall reward category reduced heterogeneity to low, explaining a substantial part of the heterogeneity (τ^2= 0.02, I^2= 28.06%), resulting in a small positive but nonsignificant pooled effect size (SMD=0.11; 95% CI, [-0.02, 0.24]). Removing one outlier[18] in overall punishment function reduced the heterogeneity to low (τ^2 = 0.05, I^2= 37.02%), which increased the overall effect size and made it significant (SMD=0.32; 95% CI, [0.10, 0.54]; eFigure 13).

There was no heterogeneity in the punishment learning/sensitivity (τ^2<0, I^2= 0.01%), appetitive Pavlovian (τ^2<0, I^2<0%), reward response vigor (τ^2=0.01, I^2=17.37%) and reward discounting (τ^2<0, I^2<0%) subcomponent. There was low-to-moderate heterogeneity in the model-based learning/flexibility (τ^2=0.032, I^2=33.40%) and risk attitude subcomponent (τ^2= 0.13, I^2=55.67%) and substantial heterogeneity in the reward learning/sensitivity (τ^2=1.30, I^2=94.70%) and aversive Pavlovian category (τ^2=0.21, I^2=72.66%). Removing one identified outlier[67] in the reward learning/sensitivity subcomponent reduced the heterogeneity somewhat (τ^2=0.13, I^2=64.95%) but did not affect the pooled effect size (SMD=0.03; 95% CI [-0.28, 0.35]). No other outliers were detected.

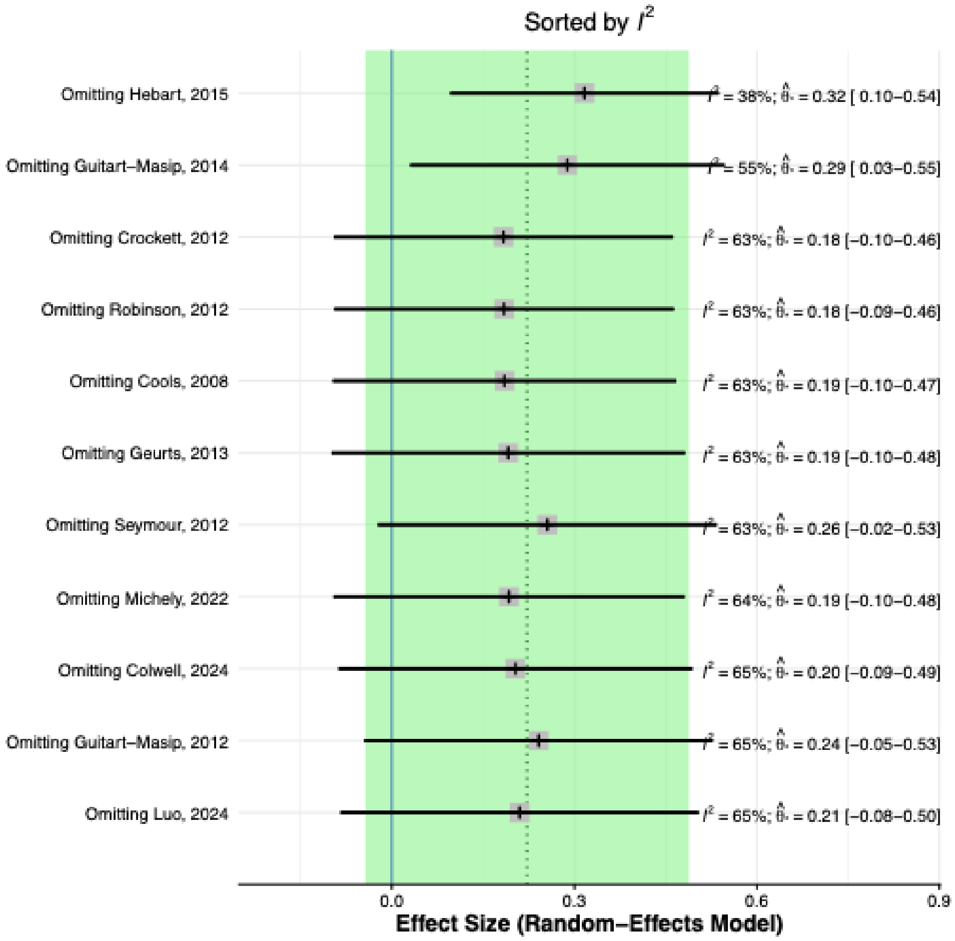

eFigure 13: Leave-one-out meta-analysis for the effect of serotonin on overall punishment function. It is ordered by heterogeneity (low to high), displaying the recalculated pooled effect size and 95% confidence interval with one study omitted each time. The green shaded area represents the 95% confidence interval of the original pooled effect size, and the dotted line represents the original estimated pooled effect size.

2.6 Moderator analyses

Several moderators were used to assess potential sources of bias in overall reward and punishment processing and subcomponents with sufficient number of studies (N>9) and substantial heterogeneity. These included reward and punishment learning/sensitivity and reward discounting components in the dopamine domain and the model-based learning/flexibility subcomponent in the serotonin domain. Task type was only assessed for the subcomponents since these would likely just recapitulate the subcomponents in the overall reward/punishment domains.

2.6.1 Dopamine

eTable 3 lists the number of studies included for all the DA moderator analyses. None of the moderators were significant predictors in the overall reward/punishment categories (proportion of female participants: reward p=0.98; punishment p=0.998; drug regimen - single vs. multiple drug administrations: reward p=0.40, not assessed in overall punishment due to too few studies with multiple drug administrations; drug type - agonist vs. antagonist: reward p=0.73; punishment p=0.75; more fine-grained drug activity classifications: reward p=0.64; punishment p=0.12; overall study age: reward p=0.91; punishment p=0.82).

Drug type was a significant predictor of dopamine in the reward discounting subcomponent (R^2= 13.88%, I^2= 6.45%, p=0.03). Studies that originally used an agonist (10 studies) had a small significant decreasing impact on reward discounting (SMD=-0.10, 95% CI [-0.16, -0.03]) compared with studies that originally used an antagonist (2 studies; note they are recoded to represent agonist), which had a small, non-significant, increasing effect on reward discounting (SMD=0.14, 95% CI [-0.06, 0.35]). It is not immediately clear what might be driving the difference between agonist and antagonist here. It is possible that this might stem from interactions with baseline dopamine levels[68] or task differences. All the antagonist studies used temporal delay discounting tasks, which we in a below exploratory analysis find are less impacted by dopamine here. This might also align with a recent theory suggesting that dopamine, particularly antagonists, might have differential effects on temporal, compared with, effort delay discounting[69] processes. However, due to the exploratory nature of this analysis, future research is needed to determine if different drug activity types do indeed have different reward discounting effects, particularly given the limited number of antagonist studies included here.

Drug type did not moderate reward or punishment learning/sensitivity (reward: p=0.60; punishment: p=0.92). More fine-grained drug type (reward: p=0.48; punishment: p=0.24; reward discounting: p=0.40), age (reward: p=0.41; too few punishment and reward discounting

studies to assess) or proportion of female participants (reward: p=0.76; punishment: p=0.997; reward discounting: p=0.17) did not moderate the reward/punishment learning/sensitivity or reward discounting results. There was only one study in the reward and punishment learning/sensitivity subcomponents that used multiple drug administrations and none in the reward discounting subcomponent. This moderator was therefore not evaluated here.

Task type did not moderate reward (p=0.26) or punishment learning/sensitivity (p=0.41). As an additional exploratory analysis, we examined if the results in the reward discounting category might differ depending on the task type. This was explored based on previous reviews suggesting that dopamine might differentially affect effort and temporal discounting[69]. Although effort versus temporal delay discounting studies were not significantly different from each other (p=0.09), the effect of dopamine on reward discounting was only significant in the effort discounting paradigms (SMD=-0.28, 95% CI [-0.52, -0.03]; 3 studies) but not in the temporal delay discounting tasks (SMD=-0.06, 95% CI [-0.13, 0.02]; 9 studies). However, due to the lack of significant difference of dopamine between these tasks, we do not interpret this further but suggest this as an area for future research to resolve.

**Moderator**	**Overall reward**	**Overall punishment**	**Reward learning/sensitivity**	**Punishment learning/sensitivity**	**Reward discounting**
Drug type	DA+=32<br>DA-=17	DA+=11<br>DA-=6	DA+=14<br>DA-=11	DA+=8<br>DA-=5	DA+=10<br>DA-=2
Specific drug type	D2-=16<br>DAP=11<br>DARI=11,<br>other=11	D2-=6<br>other=11	D2-=11<br>DAP=4<br>DARI=6<br>other=4	D2-=6<br>other=7	D2-=3<br>DAP=2<br>DARI=5<br>other=2
Task type	-	-	PILT=10<br>PST=5<br>Other=10	PILT/Other=8<br>PST=5	DD=9<br>Effort=3
Drug regimen	multiple=3<br>single=46	multiple=1<br>single=16	multiple=1<br>single=24	multiple=1<br>single=12	single=12
Age	N=34	N=10	N=16	N=8	N=8
% Female	N=41	N=13	N=20	N=11	N=10

eTable 3: Number of studies for each moderator in the dopamine (DA) domain. DA+: agonist, DA-: antagonist, D2-: D2-receptor antagonist, DAP: DA precursor, DARI: DA reuptake inhibitor, PILT: Probabilistic instrumental learning task, PST: Probabilistic selection task, DD: delay discounting task, Effort: effort discounting task

2.6.2 Serotonin

eTable 4 describes the number of studies included for all the serotonin moderator analyses. A subgroup analysis indicated that drug type significantly moderated the effect sizes in overall reward processing (R^2=17.82%, I^2=88.49%, p=0.03). However, further analysis showed that the

individual effect sizes for both the antagonist (SMD=0.26, 95% CI [-0.12, 0.65]) and agonist (SMD=-0.48, 95% CI [-1.03, 0.06]) subgroups were not significant, indicating that the overall significant moderation effect is driven by differences in directionality rather than by significant effects within each group. This effect is likely driven by the previously identified outlier[67]. Removing this outlier, drug activity is no longer a significant predictor (p=0.13).

The same was true for drug type moderating effect sizes in the model-based/flexibility subcategory (R^2=39.04%, I^2=22.92%, p=0.046), with neither the agonist (SMD=-0.19, 95% CI [-0.49, 0.11]) or antagonist (SMD=0.19, 95% CI [-0.03, 0.42]) subgroup having a significant overall effect on model-based learning. Drug type was not a significant moderator of the effect of serotonin on overall punishment function (p=0.76). Subgroup analyses with more fine-grained drug activity was not conducted since these included virtually the same groupings as the agonist/antagonist subgroup analysis. Drug regimen (single vs multiple drug administrations) did not significantly moderate the overall reward (p=0.53) or punishment (p=0.42) processes. There was only one study with multiple drug administrations in the model-based/flexibility subcomponent and thus this moderator was not assessed here. Neither age nor proportion of female participants were significant predictors in the overall reward category (age: p=0.80, %female participants: p=0.33) or in the model-based/flexibility subcomponent (too few studies to examine age; %female participants: p=0.27). There were too few studies to examine age and %female participants in the overall punishment category. Task type was not a significant moderator of model-based learning/flexibility (p=0.11).

Moderator	Overall reward	Overall punishment	Model-based learning/flexibility
Drug type	5HT+=7<br>5HT-=14	5HT+=5<br>5HT-=6	5HT+=4<br>5HT-=6
Task type	-	-	two-step=3<br>reversal=6<br>other=1
Drug regimen	multiple=2<br>single=19	multiple=2<br>single=9	multiple=1<br>single=9
Age	N=15	N=7	N=9
% Female	N=17	N=6	N=10

eTable 4: Number of studies for each moderator in the serotonin (5HT) domain. 5HT+: agonist, 5HT-: antagonist.

2.7 Publication bias

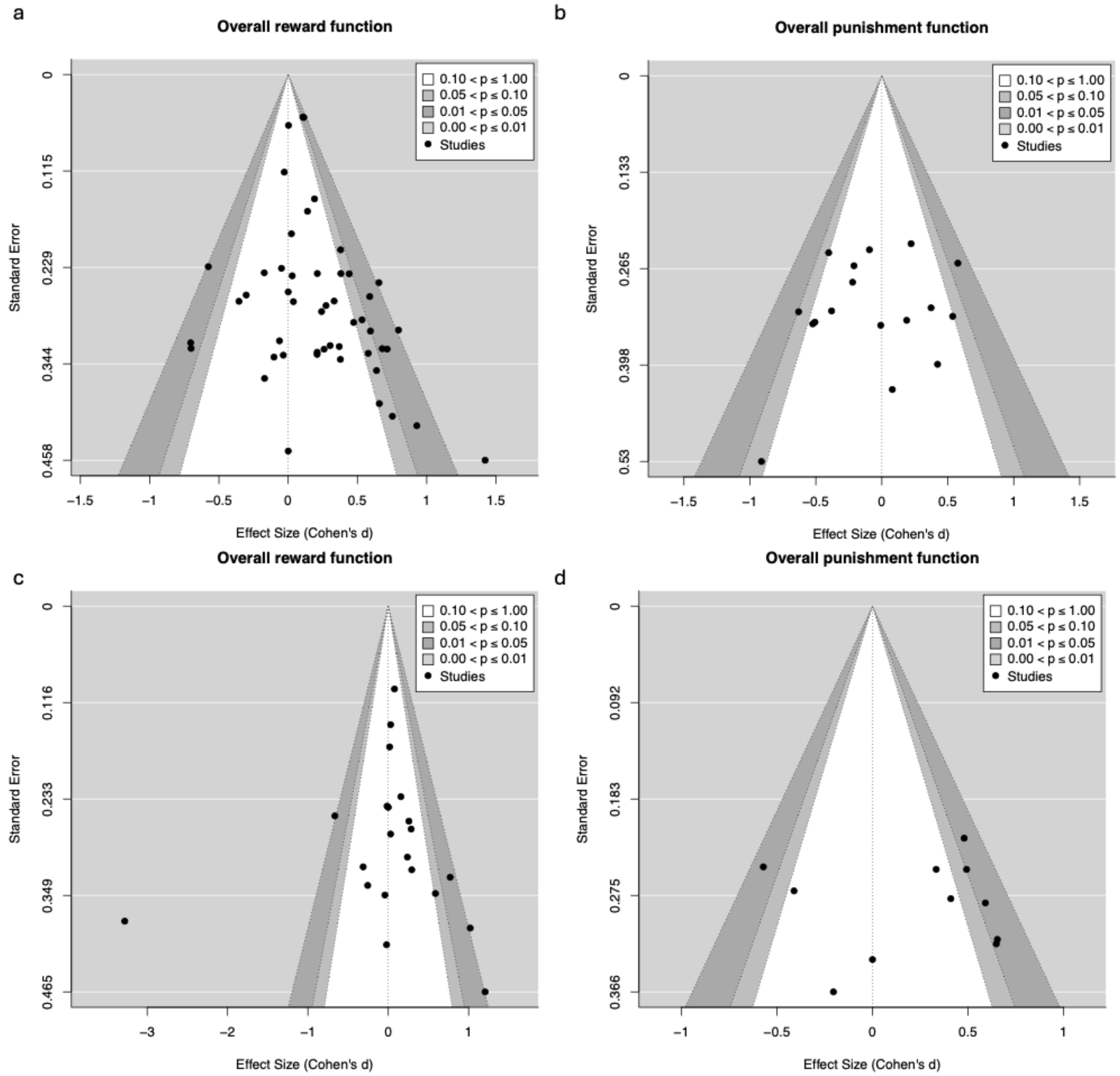

eFigure 14: Funnel plots of overall reward and dopamine function. Funnel plots of dopamine on a) overall reward function, b) overall punishment function, and funnel plots of serotonin on c) overall reward function, and d) overall punishment function.

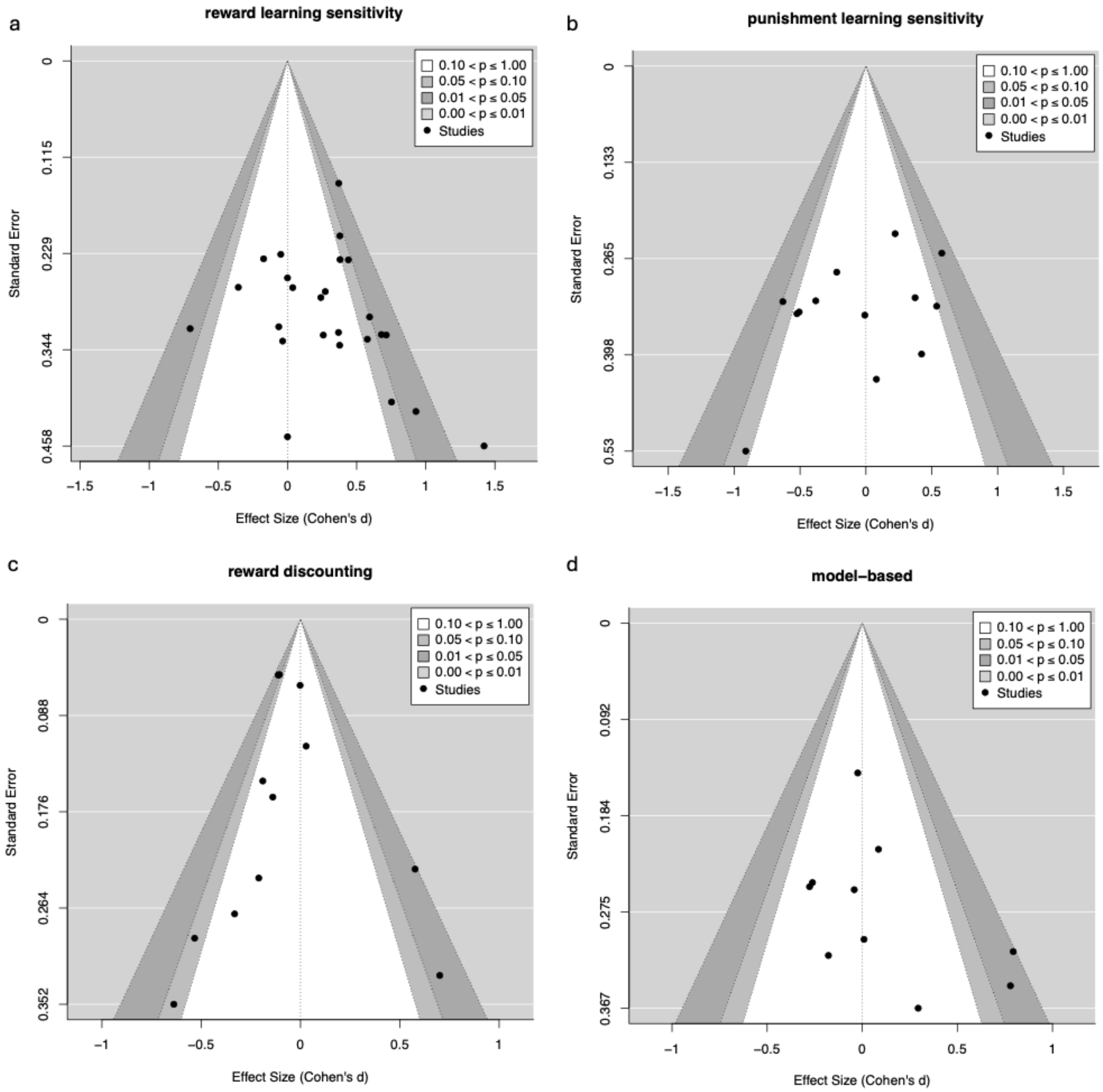

eFigure 15: Funnel plots of subcomponents. Funnel plots of dopamine on a) reward learning/sensitivity, b) punishment learning/sensitivity, c) reward discounting, and funnel plots of serotonin on d) model-based learning/flexibility.

2.8 Additional supplementary analyses

We note that olanzapine has a more complex drug activity profile than the other drug compounds included in the dopamine reward response vigor subcategory. Given the broader receptor activity profile of olanzapine, we performed a sensitivity analysis excluding this study to ensure it did not unduly influence the vigor results. The exclusion of this study[45] did not substantially alter the overall findings or conclusions (SMD = 0.30, 95% CI [0.07, 0.52]).

We categorized discounting tasks in line with previous meta-analyses where they are referred to as option valuation and categorized within the overall reward function[70,71]. This decision was further based on the fact that reward discounting paradigms typically involve rewards represented as points or money, aligning with other reward-processing tasks in this category. In contrast, tasks in the overall punishment function category primarily involve explicit losses of points or money, whereas discounting tasks incorporate aversive elements such as time delays or effort costs. Because reward discounting is typically interpreted in terms of how much a reward is valued rather than as a direct punishment process, we believe that this categorization provides a clearer theoretical interpretation. However, we recognize that the classification of reward discounting tasks is not entirely straightforward and could also be considered part of an aversive process. Grouping reward discounting as a punishment processing task, resulted in a small, non-meaningful, negative dopamine effect on overall punishment function (SMD=-0.07, 95%CI [-0.17, 0.03]), and a non-meaningful positive serotonin effect on overall punishment function (SMD=0.09, 95%CI [-0.16, 0.33]).

Study	Category	Drug, Dose	N drug	N placebo	Original design	Final design	Task	Outcome	Study Age	Study Female Proportion	Drug regimen
Addicott, 2021[72]	R L/S	methylphenidate 40mg	22	22	w	w	restless bandit	learning rate	27.7	0.57	1
Admon, 2017*[73]	R L/S	amisulpride 50mg	20	18	bw	bw	PST	choose A	25.9	0.77	1
Bellebaum, 2017[1]	R L/S	modafinil 200mg	16	20	bw	bw	PST	choose A	24.85	0	1
Bernacer, 2013[2]	R L/S	methamphetamine 0.3mg/kg	17	17	w	w	PILT	learning rate	25.3	0.41	1
Chakroun, 2023[74]	R L/S	L-dopa 150mg+37.5mg	31	31	w	w	PILT	accuracy	26.85	0	1
Cox, 2015[7]	R L/S	Phe / Tyr depletion 100g	10	10	w†	bw	PST	choose A/C	NA	NA	1
Cremer, 2023[75]	R L/S	amisulpride 400mg	23	22	bw	bw	foraging	learning rate	NA	0.47	1
Diederen, 2017[76]	R L/S	sulpiride 600mg	19	19	bw	bw	observational reversal	learning rate	24.4	0.47	1
Eisenegger, 2014[13]	R L/S	sulpiride 800mg	41	35	bw	bw	PILT	learning rate	NA	0	1
Frank, 2006*[14]	R L/S	haloperidol 2mg	19	20	w†	bw	PST	choose A	21	0.54	1
Grob, 2012[77]	R L/S	AMPT 40mg/kg	28	28	w†	bw	PRT	response bias block 3	25.8	1	1
Haarsma, 2021[78]	R L/S	sulpiride 600mg	20	20	bw	bw	reward prediction	accuracy	24.35	0.42	1
Halahakoon, 2024[79]	R L/S	pramipexole 1mg/day	21	19	bw	bw	PILT	accuracy change pre-post intervention	23.5	0.5	>1
Howlett, 2017[80]	R L/S	methylphenidate 40mg	19	19	w	w	random dot based PILT	learning rate	NA	0	1
Jansen, 2023[81]	R L/S	L-dopa 100mg+25mg	30	30	w†	bw	PILT	learning rate	22.8	0	1
Jocham, 2011*[53]	R L/S	amisulpride 200mg	16	16	w	w	PST	p(correct) win-win	26.13	0	1
Jocham, 2014[19]	R L/S	amisulpride 400mg	22	22	w	w	PILT	final accuracy	NA	0	1
Kahnt, 2015[82]	R L/S	amisulpride 400mg	27	24	bw	bw	noninstrumental outcome prediction	learning rate	22.4	0	1
Mikus, 2022[24]	R L/S	amisulpride 400mg	38	35	bw	bw	Two-step task	inverse temperature	NA	NA	1
Murray, 2019[83]	R L/S	amisulpride 400mg	18	18	w†	bw	PILT	number correct choice	32.1	0.17	1
Pessiglione, 2006[26]	R L/S	L-dopa 100mg+25mg	13	13	bw	bw	PILT	accuracy	NA	NA	1
Pizzagalli, 2008*[27]	R L/S	pramipexole 0.5mg	11	13	bw	bw	PRT	response bias block 3-1	NA	NA	1
Walsh, 2018[84]	R L/S	bupropion 150mg	20	20	bw	bw	PILT	points won	23.82	0.5	1
Weis, 2012[85]	R L/S	L-dopa 100mg+35mg	27	28	bw	bw	appetitive instrumental conditioning	accuracy learning	28.5	0.31	1
Westbrook, 2024[48]	R L/S	methylphenidate 20mg	95	97	w†	bw	PILT WM	late accuracy	NA	NA	1
Addicott, 2021[72]	R S	methylphenidate 40mg	22	22	w	w	restless bandit	inverse temperature	27.7	0.57	1
Bernacer, 2013[2]	R S	methamphetamine 0.3mg/kg	17	17	w†	bw	PILT	temperature	25.3	0.41	1
Cremer, 2023[75]	R S	amisulpride 400mg	23	22	bw	bw	foraging	inverse temperature	NA	0.47	1
Eisenegger, 2014[13]	R S	sulpiride 800mg	41	35	bw	bw	PILT	temperature	NA	0	1
Admon, 2017*[73]	P L/S	amisulpride 50mg	20	18	bw	bw	PST	avoid B	25.9	0.77	1
Bellebaum, 2017[1]	P L/S	modafinil 200mg	16	20	bw	bw	PST	avoid B	24.85	0	1
Bernacer, 2013[2]	P L/S	methamphetamine 0.3mg/kg	17	17	w†	bw	PILT	learning rate	25.3	0.41	1
Cox, 2015[7]	P L/S	Phe / Tyr depletion 100g	10	10	w	w	PST	avoid B/D	NA	NA	1

Eisenegger, 2014[13]	P L/S	sulpiride 800mg	41	35	bw	bw	PILT	learning rate	NA	0	1
Frank, 2006*[14]	P L/S	haloperidol 2mg	19	20	w†	bw	PST	avoid B	21	0.54	1
Halahakoon, 2024[79]	P L/S	pramipexole 1mg/day	21	19	bw	bw	PILT	accuracy change pre-post intervention	23.5	0.5	>1
Jocham, 2011*[53]	P L/S	amisulpride 200mg	16	16	w	w	PST	p(correct) lose-lose	26.13	0	1
Jocham, 2014[19]	P L/S	amisulpride 400mg	22	22	w	w	PILT	final accuracy	NA	0	1
Kelm, 2015[86]	P L/S	Phe / Tyr depletion 68.7g	16	16	w	w	PAL	passive avoidance errors	NA	0.31	1
Murray, 2019[83]	P L/S	amisulpride 400mg	18	18	w†	bw	PILT	accuracy	32.1	0.17	1
Pessiglione, 2006[26]	P L/S	L-dopa 100mg+25mg	13	13	bw	bw	PILT	accuracy	NA	NA	1
Walsh, 2018[84]	P L/S	bupropion 150mg	20	20	bw	bw	PILT	points lost	23.82	0.5	1
Bernacer, 2013[2]	P S	methamphetamine 0.3mg/kg	17	17	w†	bw	PILT	temperature	25.3	0.41	1
Eisenegger, 2014[13]	P S	sulpiride 800mg	41	35	bw	bw	PILT	temperature	NA	0	1
Guitart-Masip, 2012[16]	P+	L-dopa 150mg+37.5mg	16	20	bw	bw	go/nogo	nogo to win	23.15	0.34	1
Guitart-Masip, 2014[17]	P+	L-dopa 150mg+37.5mg	30	29	bw	bw	go/nogo	nogo to win	NA	NA	1
Hebart, 2015[18]	P+	Phe / Tyr depletion	35	34	bw	bw	PIT	appetitive PIT	NA	NA	1
Scholz, 2022[32]	P+	tolcapone 200mg	35	35	w†	bw	go/nogo	nogo to win	31.3	0.26	1
Weber, 2016[47]	P+	amisulpride 400mg	41	40	bw	bw	PIT	button press rew-unrew CS	21.8	NA	1
Guitart-Masip, 2012[16]	P-	L-dopa 150mg+37.5mg	16	20	bw	bw	go/nogo	go to avoid	23.15	0.34	1
Guitart-Masip, 2014[17]	P-	L-dopa 150mg+37.5mg	30	29	bw	bw	go/nogo	go to avoid	NA	NA	1
Hebart, 2015[18]	P-	Phe / Tyr depletion	35	34	bw	bw	PIT	aversive PIT	NA	NA	1
Scholz, 2022[32]	P-	tolcapone 200mg	35	35	w†	bw	go/nogo	go to avoid	31.3	0.26	1
Daood, 2022[87]	RD	methylphenidate 20mg	57	57	w	w	DD	p(choose now)	26.42	0.6	1
Erfanian Abdoust, 2024*[88]	RD	haloperidol 2mg	62	62	w	w	DD	p(high cost option)	22.79	0.52	1
Kayser, 2012[89]	RD	tolcapone 200mg	23	23	w	w	DD	p(choose now)	NA	0.56	1
Kelm, 2013[90]	RD	Phe / Tyr depletion 68.7g	15	15	w	w	DD	p(choose now)	NA	0	1
Pine, 2010[65]	RD	L-dopa 150mg	13	13	w	w	DD	k parameter	21	0.57	1
Smith, 2024[91]	RD	L-dopa 150mg	76	76	w†	bw	DD	Larger-but-later choices	28.26	0.42	1
Soder, 2021[34]	RD	d-amphetamine 20mg	28	28	w†	bw	physical effort	p(hard task)	24.07	0.5	1
Wagner, 2020*[92]	RD	haloperidol 2mg	23	26	bw	bw	DD	p(delay)	23.85	0.74	1
Wardle, 2011[37]	RD	d-amphetamine 20mg	17	17	w†	bw	physical effort	p(hard task)	22.7	0.65	1
Weber, 2016[47]	RD	amisulpride 400mg	40	40	bw	bw	DD	p(choose now)	NA	NA	1
Westbrook, 2020[38]	RD	methylphenidate 20mg	46	46	w	w	cognitive effort	subjective value(hard task)	NA	NA	1
de Wit, 2002[93]	RD	d-amphetamine 20mg	36	36	w†	bw	DD	k parameter	24	0.5	1
Abler, 2007[45]	RV	olanzapine 5mg	8	8	w	w	MID	RT high reward	31.3	0.5	1

Au-Yeung, 2024[94]	RV	pramipexole 1mg/day	19	18	bw	bw	eye-tracking vigor	Velocity residuals	23.59	0.48	>1
Beierholm, 2013[95]	RV	L-dopa 150mg+37.5mg	30	30	bw	bw	odd-ball discrimination	average reward rate on RTs	24.15	0.43	1
Graf, 2018[96]	RV	amisulpride 200mg/day	17	17	w	w	MID	RT 100-0	23.8	0	>1
Grimm, 2021[97]	RV	L-dopa 100mg+25mg	45	45	w	w	MID	RT high-neutral reward	22.81	0.51	1
Ikeda, 2019[98]	RV	bupropion 150mg	15	15	w	w	MID	RT high-neutral reward	31.7	0.53	1
Mehta, 2001[99]	RV	bromocriptine 1.25mg	18	18	w	w	Reward-responsivity	No. cards sorted in sort 3 - No. cards sorted in sorts 2/4	NA	0	1
Mehta, 2005[100]	RV	Phe / Tyr depletion	14	14	w	w	Reward-responsivity	No. extra cards correctly sorted	33.9	0.14	1
Michely, 2020[46]	RV	L-dopa 150mg	20	20	w	w	dynamic effort	grip force high reward	25.6	0.45	1
Clatworthy, 2009[101]	MB/F	methylphenidate 60mg	9	9	w	w	reversal	reversal learning	NA	0	1
Deserno, 2021[10]	MB/F	L-dopa 150mg+37.5mg	62	62	w	w	model-based bandit	model-based choice	NA	NA	1
Ersche, 2011[102]	MB/F	amisulpride 400mg	18	18	w	w	probabilistic reversal	perseverative errors	32.7	0.2	1
Janssen, 2015[103]	MB/F	sulpiride 400mg	22	22	w	w	observational reversal	reversal errors	32.2	0	1
Mikus, 2022[24]	MB/F	amisulpride 400mg	38	35	bw	bw	Two-step task	omega parameter	NA	NA	1
Robinson, 2010[29]	MB/F	Phe / Tyr depletion	27	27	w	w	observational reversal	reversal errors	NA	0.48	1
Vo, 2018[104]	MB/F	L-dopa 100mg+25mg	13	13	bw	bw	probabilistic reversal	reversal errors	NA	NA	1
Wunderlich, 2012[40]	MB/F	L-dopa 150mg+37.5mg	18	18	w	w	Two-step task	omega parameter	23.3	0	1
van den Bosch, 2022[105]	MB/F	methylphenidate 20mg	88	88	w†	bw	observational reversal	accuracy reversal	NA	NA	1
van der Schaaf, 2013[106]	MB/F	methylphenidate 20mg	19	19	w	w	observational reversal	accuracy reversal	20.9	0.52	1
van der Schaaf, 2014[107]	MB/F	sulpiride 400mg	23	23	w	w	observational reversal	accuracy reversal	NA	0.5	1
Burke, 2018[66]	RA	amisulpride 400mg	45	48	bw	bw	gambling	utility curvature	NA	NA	1
Campbell-Meiklejohn, 2011*[108]	RA	pramipexole 0.176µg	15	15	bw	bw	gambling	p(gamble)	24.9	0.53	1
Hirschbichler, 2022[109]	RA	L-dopa 100mg	30	30	w	w	gambling	p(gamble)	31.67	0.53	1
Norbury, 2013[110]	RA	cabergoline 1.5mg	20	20	w	w	gambling	p(gamble)	26.7	NA	1
Riba, 2008*[111]	RA	pramipexole 0.5mg	15	15	w	w	gambling	p(risky choice)	24.4	NA	1
Rigoli, 2016[112]	RA	L-dopa 150mg+37.5mg	32	32	w	w	gambling	p(gamble)	23.4	0.5	1
Rutledge, 2015[113]	RA	L-dopa 150mg+37.5mg	30	30	w	w	gambling	p(gamble) gain	23.4	0.63	1

eTable 5: Characteristics of all dopamine studies. Asterisks indicate studies that used a single low dose of an agonist or antagonists. These effects were interpreted contrary to their typical activity profile (e.g., a low-dose agonist acting as an antagonist), as suggested by the literature. Daggers indicate studies that were originally within-subject studies (in column ’Original design’) but we could only calculate a between-subjects Cohen’s *d* (indicated in column ’Final design’). In drug regimen, 1 indicates a single drug administration and >1 indicates

multiple drug administrations. Abbreviations: R L/S: reward learning/sensitivity; R S: reward sensitivity; P L/S: punishment learning/sensitivity; P S: punishment sensitivity; P+: appetitive Pavlovian; P-: aversive Pavlovian; RD: reward discounting; RV: reward vigor: MB/F: model-based learning/flexibility; RA: risk attitude; w: within-subject studies; bw: between-subject studies; PST: probabilistic selection task; PILT: probabilistic instrumental learning task; WM: working memory; PAL: passive avoidance learning; PIT: Pavlovian instrumental task; DD: delay discounting; MID: monetary incentive delay.

Study	Category	Drug, Dose	N drug	N placebo	Original design	Final design	Task	Outcome	Study Age	Study Female Proportion	Drug regimen
Colwell, 2024[114]	R L/S	fenfluramine 15mg b.i.d.	26	27	bw	bw	PILT	learning rate	20.17	0.6	>1
Cools, 2008[6]	R L/S	TD	12	12	w	w	observational reversal	non-reversal errors	22.4	0.67	1
Finger, 2007[115]	R L/S	TD	16	19	bw	bw	PAL	omission errors	27.94	0.52	1
Gilger, 2024[116]	R L/S	TD	98	98	w†	bw	Two-step	main effect reward	32.2	0.38	1
Jepma, 2010[117]	R L/S	citalopram 30mg	16	17	bw	bw	restless bandit	inverse temperature	21.55	0.49	1
Luo, 2024[67]	R L/S	escitalopram 20mg	32	33	bw	bw	probabilistic reversal	learning rate	26	0.49	1
Michely, 2022[118]	R L/S	citalopram 20mg/day	33	33	bw	bw	gambling card	learning rate	24.65	0.39	>1
Robinson, 2012[28]	R L/S	TD	21	20	bw	bw	observational reversal	non-reversal errors	27.6	1	1
Seymour, 2012[33]	R L/S	TD	15	15	bw	bw	restless bandit	sensitivity	NA	NA	1
Colwell, 2024[114]	R S	fenfluramine 15mg b.i.d.	26	27	bw	bw	PILT	outcome sensitivity	20.17	0.6	>1
Colwell, 2024[114]	P L/S	fenfluramine 15mg b.i.d.	26	27	bw	bw	PILT	learning rate	20.17	0.6	>1
Luo, 2024[67]	P L/S	escitalopram 20mg	32	33	bw	bw	probabilistic reversal	learning rate	26	0.49	1
Michely, 2022[118]	P L/S	citalopram 20mg/day	33	33	bw	bw	gambling card	learning rate	24.65	0.39	>1
Seymour, 2012[33]	P L/S	TD	15	15	bw	bw	restless bandit	sensitivity	NA	NA	1
Colwell, 2024[114]	P S	fenfluramine 15mg b.i.d.	26	27	bw	bw	PILT	outcome sensitivity	20.17	0.6	>1
Geurts, 2013[15]	P+	TD	45	45	w	w	PIT	appetitive PIT	23.8	NA	1
Guitart-Masip, 2012[16]	P+	citalopram 24mg oral	16	20	bw	bw	go/nogo	nogo to win	23.3	0.49	1
Guitart-Masip, 2014[17]	P+	citalopram 30mg	27	29	bw	bw	go/nogo	nogo to win	NA	NA	1
Hebart, 2015[18]	P+	TD	34	34	bw	bw	PIT	appetitive PIT	NA	NA	1
Cools, 2008[6]	P-	TD	12	12	w	w	observational reversal	non-reversal errors	22.4	0.67	1
Crockett, 2009[9]	P-	TD	22	22	w	w	go/nogo RT	punishment-induced slowing	NA	NA	1
Crockett, 2012[8]	P-	TD	24	24	w	w	go/nogo RT	punishment-induced slowing	NA	NA	1
Gaber, 2015[119]	P-	TD	24	24	w	w	go/nogo RT	punishment-induced slowing	25.34	NA	1
Geurts, 2013[15]	P-	TD	45	45	w	w	PIT	aversive PIT	23.8	NA	1
Guitart-Masip, 2012[16]	P-	citalopram 24mg oral	16	20	bw	bw	go/nogo	go to avoid	23.3	0.49	1

Guitart-Masip, 2014[17]	P-	citalopram 30mg	27	29	bw	bw	go/nogo	go to avoid	NA	NA	1
Hebart, 2015[18]	P-	TD	34	34	bw	bw	PIT	aversive PIT	NA	NA	1
Helmbold, 2015[120]	P-	TD	18	18	w	w	go/nogo RT	punishment-induced slowing	24.22	NA	1
Robinson, 2012[28]	P-	TD	21	20	bw	bw	observational reversal	non-reversal errors	27.6	1	1
Crean, 2002[121]	RD	TD	20	20	w†	bw	DD	k parameter	21.5	0	1
Schweighofer, 2008[122]	RD	TD	20	20	w	w	DD dynamic	discount factor gamma	NA	0	1
Tanaka, 2007[123]	RD	TD	12	12	w†	bw	multi-step DD	p(large reward choices)	NA	0	1
Worbe, 2014[124]	RD	TD	22	22	bw	bw	DD	k parameter	28.97	0.61	1
Beierholm, 2013[95]	RV	citalopram 24mg oral	30	30	bw	bw	odd-ball discrimination	average reward rate on RTs	23.91	0.5	1
Cools, 2005[125]	RV	TD	10	12	bw	bw	CRRT	RT 90-10	23.55	0	1
Roiser, 2006[126]	RV	TD	30	30	w	w	CRRT	RT 90-10	26.75	0.43	1
Steding, 2023[127]	RV	TD	24	24	w	w	physical effort	button press high-low	NA	1	1
Chamberlain, 2006[128]	MB/F	citalopram 30mg	20	20	bw	bw	reversal	perseverative errors	25.82	0.5	1
Cools, 2008[6]	MB/F	TD	12	12	w	w	observational reversal	switch errors	22.4	0.67	1
Evers, 2004[129]	MB/F	TD	15	15	w†	bw	probabilistic reversal	perseverative errors	21.8	0.8	1
Gilger, 2024[116]	MB/F	TD	98	98	w†	bw	Two-step	omega parameter	32.2	0.38	1
Kanen, 2020[130]	MB/F	TD	30	32	bw	bw	probabilistic reversal	perseveration	NA	0.47	1
Langley, 2023[131]	MB/F	escitalopram 20mg /day	32	34	bw	bw	Two-step task	Reward × Transition	25	65.17	>1
Skandali, 2018[132]	MB/F	escitalopram 20mg	31	33	bw	bw	Probabilistic reversal	reversal errors	26	0.49	1
Thirkettle, 2019[133]	MB/F	Tryptophan 0.8g	22	22	bw	bw	probabilistic reversal	reversal errors	21.1	0.64	1
Worbe, 2015[134]	MB/F	TD	18	18	bw	bw	3-stage instrumental learning	devalued choices	26.33	0.56	1
Worbe, 2016[39]	MB/F	TD	22	22	bw	bw	Two-step task	omega parameter	29.14	0.52	1
Anderson, 2003[135]	RA	TD	15	13	bw	bw	gambling	mean indifference odds against win	23	0.39	1
Campbell-Meiklejohn, 2011[108]	RA	TD	17	17	bw	bw	gambling	p(gamble)	24.18	0.53	1
Faulkner, 2017[136]	RA	TD	26	26	w	w	gambling	p(gamble)	29.45	0.54	1
Macoveanu, 2014[137]	RA	fluoxetine 20/40mg/day	13	16	bw	bw	gambling	p(risky choice)	NA	0	>1
Murphy, 2009[138]	RA	l-tryptophan 3g/day	15	15	bw	bw	gambling	p(gamble)	25.54	0.53	>1
Rogers, 2003[111]	RA	TD	18	18	bw	bw	gambling	p(gamble)	23.8	0.5	1

eTable 6: Characteristics of all serotonin studies. Daggers indicate studies that were originally within-subject studies (in column 'Original design') but we could only calculate a between-subjects Cohen's *d* (indicated in column 'Final design'). In drug regimen, 1 indicates a single drug administration and >1 indicates multiple drug administrations. Abbreviations: R L/S: reward

learning/sensitivity; R S: reward sensitivity; P L/S: punishment learning/sensitivity; P S: punishment sensitivity; P+: appetitive Pavlovian; P-: aversive Pavlovian; RD: reward discounting; RV: reward vigor: MB/F: model-based learning/flexibility; RA: risk attitude; w: within-subject studies; bw: between-subject studies; PST: probabilistic selection task; PILT: probabilistic instrumental learning task; PAL: passive avoidance learning; PIT: Pavlovian instrumental task; DD: delay discounting; MID: monetary incentive delay; CRRT: cued-reinforcement reaction-time task.

3 References

1. Bellebaum C, Kuchinke L, Roser P. Modafinil alters decision making based on feedback history - a randomized placebo-controlled double blind study in humans. https://doi.org/10.1177/0269881116668591. *Journal of psychopharmacology (Oxford, England)*. 2017;31(2):243-249.
2. Bernacer J, Corlett PR, Ramachandra P, et al. Methamphetamine-induced disruption of frontostriatal reward learning signals: relation to psychotic symptoms. https://doi.org/10.1176/appi.ajp.2013.12070978. *The American journal of psychiatry*. 2013;170(11):1326-34.
3. Chakroun K, Mathar D, Wiehler A, Ganzer F, Peters J. Dopaminergic modulation of the exploration/exploitation trade-off in human decision-making. https://doi.org/10.7554/eLife.51260. *eLife*. 2020;9
4. Cook JL, Swart JC, Frobose MI, et al. Catecholaminergic modulation of meta-learning. https://doi.org/10.7554/eLife.51439. *eLife*. 2019;8
5. Cools R, Frank MJ, Gibbs SE, Miyakawa A, Jagust W, D'Esposito M. Striatal dopamine predicts outcome-specific reversal learning and its sensitivity to dopaminergic drug administration. https://doi.org/10.1523/JNEUROSCI.4467-08.2009. *The Journal of neuroscience : the official journal of the Society for Neuroscience*. 2009;29(5):1538-43.
6. Cools R, Robinson OJ, Sahakian B. Acute tryptophan depletion in healthy volunteers enhances punishment prediction but does not affect reward prediction. https://doi.org/10.1038/sj.npp.1301598. *Neuropsychopharmacology : official publication of the American College of Neuropsychopharmacology*. 2008;33(9):2291-9.
7. Cox SM, Frank MJ, Larcher K, et al. Striatal D1 and D2 signaling differentially predict learning from positive and negative outcomes. https://doi.org/10.1016/j.neuroimage.2014.12.070. *Neuroimage*. 2015;109:95-101.
8. Crockett MJ, Clark L, Apergis-Schoute AM, Morein-Zamir S, Robbins TW. Serotonin modulates the effects of Pavlovian aversive predictions on response vigor. https://doi.org/10.1038/npp.2012.75. *Neuropsychopharmacology : official publication of the American College of Neuropsychopharmacology*. 2012;37(10):2244-52.
9. Crockett MJ, Clark L, Robbins TW. Reconciling the role of serotonin in behavioral inhibition and aversion: acute tryptophan depletion abolishes punishment-induced inhibition in humans. https://doi.org/10.1523/JNEUROSCI.2513-09.2009. *The Journal of neuroscience : the official journal of the Society for Neuroscience*. 2009;29(38):11993-9.
10. Deserno L, Moran R, Michely J, Lee Y, Dayan P, Dolan RJ. Dopamine enhances model-free credit assignment through boosting of retrospective model-based inference. https://doi.org/10.7554/eLife.67778. *eLife*. 2021;10
11. Dodds CM, Muller U, Clark L, van Loon A, Cools R, Robbins TW. Methylphenidate has differential effects on blood oxygenation level-dependent signal related to cognitive subprocesses of reversal learning. https://doi.org/10.1523/JNEUROSCI.1153-08.2008. *The Journal of neuroscience : the official journal of the Society for Neuroscience*. 2008;28(23):5976-82.

12. Dubois M, Habicht J, Michely J, Moran R, Dolan RJ, Hauser TU. Human complex exploration strategies are enriched by noradrenaline-modulated heuristics. https://doi.org/10.7554/eLife.59907. *eLife*. 2021;10

13. Eisenegger C, Naef M, Linssen A, et al. Role of dopamine D2 receptors in human reinforcement learning. https://doi.org/10.1038/npp.2014.84. *Neuropsychopharmacology : official publication of the American College of Neuropsychopharmacology*. 2014;39(10):2366-75.

14. Frank MJ, O'Reilly RC. A mechanistic account of striatal dopamine function in human cognition: psychopharmacological studies with cabergoline and haloperidol. https://doi.org/10.1037/0735-7044.120.3.497. *Behavioral neuroscience*. 2006;120(3):497-517.

15. Geurts DE, Huys QJ, den Ouden HE, Cools R. Serotonin and aversive Pavlovian control of instrumental behavior in humans. https://doi.org/10.1523/jneurosci.2749-13.2013. *J Neurosci*. 2013;33(48):18932-9.

16. Guitart-Masip M, Chowdhury R, Sharot T, Dayan P, Duzel E, Dolan RJ. Action controls dopaminergic enhancement of reward representations. https://doi.org/10.1073/pnas.1202229109. *Proceedings of the National Academy of Sciences of the United States of America*. 2012;109(19):7511-6.

17. Guitart-Masip M, Economides M, Huys QJ, et al. Differential, but not opponent, effects of L -DOPA and citalopram on action learning with reward and punishment. https://doi.org/10.1007/s00213-013-3313-4. *Psychopharmacology (Berl)*. 2014;231(5):955-66.

18. Hebart MN, Glascher J. Serotonin and dopamine differentially affect appetitive and aversive general Pavlovian-to-instrumental transfer. https://doi.org/10.1007/s00213-014-3682-3. *Psychopharmacology*. 2015;232(2):437-51.

19. Jocham G, Klein TA, Ullsperger M. Differential modulation of reinforcement learning by D2 dopamine and NMDA glutamate receptor antagonism. https://doi.org/10.1523/JNEUROSCI.0757-14.2014. *The Journal of neuroscience : the official journal of the Society for Neuroscience*. 2014;34(39):13151-62.

20. Kanen JW, Ersche KD, Fineberg NA, Robbins TW, Cardinal RN. Computational modelling reveals contrasting effects on reinforcement learning and cognitive flexibility in stimulant use disorder and obsessive-compulsive disorder: remediating effects of dopaminergic D2/3 receptor agents. https://doi.org/10.1007/s00213-019-05325-w. *Psychopharmacology*. 2019;236(8):2337-2358.

21. Kayser AS, Mitchell JM, Weinstein D, Frank MJ. Dopamine, locus of control, and the exploration-exploitation tradeoff. https://doi.org/10.1038/npp.2014.193. *Neuropsychopharmacology : official publication of the American College of Neuropsychopharmacology*. 2015;40(2):454-62.

22. Kroemer NB, Lee Y, Pooseh S, Eppinger B, Goschke T, Smolka MN. L-DOPA reduces model-free control of behavior by attenuating the transfer of value to action. https://doi.org/10.1016/j.neuroimage.2018.10.075. *NeuroImage*. 2019;186:113-125.

23. Meyniel F, Goodwin GM, Deakin JW, et al. A specific role for serotonin in overcoming effort cost. https://doi.org/10.7554/eLife.17282. *Elife*. 2016;5

24. Mikus N, Korb S, Massaccesi C, et al. Effects of dopamine D2/3 and opioid receptor antagonism on the trade-off between model-based and model-free behaviour in healthy volunteers. https://doi.org/10.7554/eLife.79661. *eLife*. 2022;11

25. Ohmann HA, Kuper N, Wacker J. A low dosage of the dopamine D2-receptor antagonist sulpiride affects effort allocation for reward regardless of trait extraversion. https://doi.org/10.1017/pen.2020.7. *Personality neuroscience*. 2020;3:e7-e7.
26. Pessiglione M, Seymour B, Flandin G, Dolan RJ, Frith CD. Dopamine-dependent prediction errors underpin reward-seeking behaviour in humans. https://doi.org/10.1038/nature05051. *Nature*. 2006;442(7106):1042-5.
27. Pizzagalli DA, Evins AE, Schetter EC, et al. Single dose of a dopamine agonist impairs reinforcement learning in humans: behavioral evidence from a laboratory-based measure of reward responsiveness. https://doi.org/10.1007/s00213-007-0957-y. *Psychopharmacology*. 2008;196(2):221-32.
28. Robinson OJ, Cools R, Sahakian BJ. Tryptophan depletion disinhibits punishment but not reward prediction: implications for resilience. https://doi.org/10.1007/s00213-011-2410-5. *Psychopharmacology*. 2012;219(2):599-605.
29. Robinson OJ, Standing HR, DeVito EE, Cools R, Sahakian BJ. Dopamine precursor depletion improves punishment prediction during reversal learning in healthy females but not males. https://doi.org/10.1007/s00213-010-1880-1. *Psychopharmacology*. 2010;211(2):187-95.
30. Rostami Kandroodi M, Cook JL, Swart JC, et al. Effects of methylphenidate on reinforcement learning depend on working memory capacity. https://doi.org/10.1007/s00213-021-05974-w. *Psychopharmacology*. 2021;238(12):3569-3584.
31. Scholl J, Kolling N, Nelissen N, Browning M, Rushworth MFS, Harmer CJ. Beyond negative valence: 2-week administration of a serotonergic antidepressant enhances both reward and effort learning signals. https://doi.org/10.1371/journal.pbio.2000756. *PLoS biology*. 2017;15(2):e2000756-e2000756.
32. Scholz V, Hook RW, Kandroodi MR, et al. Cortical dopamine reduces the impact of motivational biases governing automated behaviour. https://doi.org/10.1038/s41386-022-01291-8. *Neuropsychopharmacology : official publication of the American College of Neuropsychopharmacology*. 2022;47(8):1503-1512.
33. Seymour B, Daw ND, Roiser JP, Dayan P, Dolan R. Serotonin selectively modulates reward value in human decision-making. https://doi.org/10.1523/JNEUROSCI.0053-12.2012. *The Journal of neuroscience : the official journal of the Society for Neuroscience*. 2012;32(17):5833-42.
34. Soder HE, Cooper JA, Lopez-Gamundi P, et al. Dose-response effects of d-amphetamine on effort-based decision-making and reinforcement learning. https://doi.org/10.1038/s41386-020-0779-8. *Neuropsychopharmacology : official publication of the American College of Neuropsychopharmacology*. 2021;46(6):1078-1085.
35. Tanaka SC, Shishida K, Schweighofer N, Okamoto Y, Yamawaki S, Doya K. Serotonin affects association of aversive outcomes to past actions. https://doi.org/10.1523/jneurosci.2799-09.2009. *J Neurosci*. 2009;29(50):15669-74.
36. Vo A, Seergobin KN, Morrow SA, MacDonald PA. Levodopa impairs probabilistic reversal learning in healthy young adults. https://doi.org/10.1007/s00213-016-4322-x. *Psychopharmacology*. 2016;233(14):2753-63.
37. Wardle MC, Treadway MT, Mayo LM, Zald DH, de Wit H. Amping up effort: effects of d-amphetamine on human effort-based decision-making.

https://doi.org/10.1523/JNEUROSCI.4387-11.2011. *The Journal of neuroscience : the official journal of the Society for Neuroscience*. 2011;31(46):16597-602.
38. Westbrook A, van den Bosch R, Maatta JI, et al. Dopamine promotes cognitive effort by biasing the benefits versus costs of cognitive work. https://doi.org/10.1126/science.aaz5891. *Science (New York, NY)*. 2020;367(6484):1362-1366.
39. Worbe Y, Palminteri S, Savulich G, et al. Valence-dependent influence of serotonin depletion on model-based choice strategy. https://doi.org/10.1038/mp.2015.46. *Molecular psychiatry*. 2016;21(5):624-9.
40. Wunderlich K, Smittenaar P, Dolan RJ. Dopamine enhances model-based over model-free choice behavior. https://doi.org/10.1016/j.neuron.2012.03.042. *Neuron*. 2012;75(3):418-24.
41. Glazer J, Murray CH, Nusslock R, Lee R, de Wit H. Low doses of lysergic acid diethylamide (LSD) increase reward-related brain activity. *Neuropsychopharmacology*. Jan 2023;48(2):418-426. doi:10.1038/s41386-022-01479-y
42. Kanen JW, Luo Q, Rostami Kandroodi M, et al. Effect of lysergic acid diethylamide (LSD) on reinforcement learning in humans. *Psychol Med*. Oct 2023;53(14):6434-6445. doi:10.1017/s0033291722002963
43. Pokorny T, Duerler P, Seifritz E, Vollenweider FX, Preller KH. LSD acutely impairs working memory, executive functions, and cognitive flexibility, but not risk-based decision-making. *Psychol Med*. Oct 2020;50(13):2255-2264. doi:10.1017/s0033291719002393
44. Marona-Lewicka D, Thisted RA, Nichols DE. Distinct temporal phases in the behavioral pharmacology of LSD: dopamine D2 receptor-mediated effects in the rat and implications for psychosis. *Psychopharmacology (Berl)*. Jul 2005;180(3):427-35. doi:10.1007/s00213-005-2183-9
45. Abler B, Erk S, Walter H. Human reward system activation is modulated by a single dose of olanzapine in healthy subjects in an event-related, double-blind, placebo-controlled fMRI study. https://doi.org/10.1007/s00213-006-0690-y. *Psychopharmacology (Berl)*. 2007;191(3):823-33.
46. Michely J, Viswanathan S, Hauser TU, Delker L, Dolan RJ, Grefkes C. The role of dopamine in dynamic effort-reward integration. *Neuropsychopharmacology : official publication of the American College of Neuropsychopharmacology*. 2020;45:1448-1453.
47. Weber SC, Beck-Schimmer B, Kajdi ME, Müller D, Tobler PN, Quednow BB. Dopamine D2/3- and μ-opioid receptor antagonists reduce cue-induced responding and reward impulsivity in humans. https://doi.org/10.1038/tp.2016.113. *Transl Psychiatry*. 2016;6(7):e850-e850.
48. Westbrook A, van den Bosch R, Hofmans L, et al. Striatal Dopamine Can Enhance Learning, Both Fast and Slow, and Also Make it Cheaper. https://doi.org/10.1101/2024.02.14.580392. *bioRxiv*. 2024:2024.02.14.580392-2024.02.14.580392.
49. Daw DD, Kakade S, Dayan P. Opponent interactions between serotonin and dopamine. *Neural Networks*. 2002;15:603–616-603–616.
50. Coukell AJ, Spencer CM, Benfield P. Amisulpride. https://doi.org/10.2165/00023210-199606030-00006. *CNS Drugs*. 1996;6(3):237-256.
51. Papp M, Wieronska J. Antidepressant-like activity of amisulpride in two animal models of depression. https://doi.org/10.1177/026988110001400106. *J Psychopharmacol*. 2000;14(1):46-52.

52. Schoemaker H, Claustre Y, Fage D, et al. Neurochemical characteristics of amisulpride, an atypical dopamine D2/D3 receptor antagonist with both presynaptic and limbic selectivity. *J Pharmacol Exp Ther*. 1997;280(1):83-97.
53. Jocham G, Klein TA, Ullsperger M. Dopamine-mediated reinforcement learning signals in the striatum and ventromedial prefrontal cortex underlie value-based choices. https://doi.org/10.1523/jneurosci.3904-10.2011. *J Neurosci*. 2011;31(5):1606-13.
54. Higgins JP, Thompson SG, Deeks JJ, Altman DG. Measuring inconsistency in meta-analyses. https://doi.org/10.1136/bmj.327.7414.557. *Bmj*. 2003;327(7414):557-60.
55. Harrer M, Cuijpers P, Furukawa T, Ebert DD. dmetar: Companion R Package For The Guide 'Doing Meta-Analysis in R'. 2019;
56. Egger M, Davey Smith G, Schneider M, Minder C. Bias in meta-analysis detected by a simple, graphical test. https://doi.org/10.1136/bmj.315.7109.629. *Bmj*. 1997;315(7109):629-34.
57. Higgins JPT, Thomas J, Chandler J, et al. Cochrane Handbook for Systematic Reviews of Interventions version 6.5. 2024.
58. Higgins JPT, Altman DG, Gotzsche PC, et al. The Cochrane Collaboration's tool for assessing risk of bias in randomised trials. https://doi.org/10.1136/bmj.d5928. *BMJ*. 2011;343(oct18 2):d5928-d5928.
59. Cumming G. *Understanding the new statistics: Effect sizes, confidence intervals, and meta-analysis*. Routledge; 2013.
60. Goulet-Pelletier J-C, Cousineau D. A review of effect sizes and their confidence intervals, Part I: The Cohen's d family. https://doi.org/10.20982/tqmp.14.4.p242. *The Quantitative Methods for Psychology*. 2018;14(4):242-265.
61. Cousineau D, Goulet-Pelletier J-C. A study of confidence intervals for Cohen's dp in within-subject designs with new proposals. https://doi.org/10.20982/tqmp.17.1.p051. *The Quantitative Methods for Psychology*. 2021;17(1):51-75.
62. Lakens D. Calculating and reporting effect sizes to facilitate cumulative science: a practical primer for t-tests and ANOVAs. *Frontiers in psychology*. 2013;4:863-863.
63. Caldwell AR. Exploring Equivalence Testing with the Updated TOSTER R Package. https://doi.org/10.31234/osf.io/ty8de. *PsyArXiv*. 2022;
64. Algina J, Keselman HJ. Approximate Confidence Intervals for Effect Sizes. https://doi.org/10.1177/0013164403256358. *Educational and Psychological Measurement*. 2003;63(4):537-553.
65. Pine A, Shiner T, Seymour B, Dolan RJ. Dopamine, time, and impulsivity in humans. *The Journal of neuroscience : the official journal of the Society for Neuroscience*. 2010;30:8888-96.
66. Burke CJ, Soutschek A, Weber S, et al. Dopamine Receptor-Specific Contributions to the Computation of Value. *Neuropsychopharmacology : official publication of the American College of Neuropsychopharmacology*. 2018;43:1415-1424.
67. Luo Q, Kanen JW, Bari A, et al. Comparable roles for serotonin in rats and humans for computations underlying flexible decision-making. https://doi.org/10.1038/s41386-023-01762-6. *Neuropsychopharmacology*. 2024;49(3):600-608.
68. Cools R, D'Esposito M. Inverted-U-shaped dopamine actions on human working memory and cognitive control. https://doi.org/10.1016/j.biopsych.2011.03.028. *Biol Psychiatry*. 2011;69(12):e113-25.

69. Soutschek A, Jetter A, Tobler PN. Toward a Unifying Account of Dopamine's Role in Cost-Benefit Decision Making. https://doi.org/10.1016/j.bpsgos.2022.02.010. *Biol Psychiatry Glob Open Sci*. 2023;3(2):179-186.
70. Costello H, Berry AJ, Reeves S, et al. Disrupted reward processing in Parkinson's disease and its relationship with dopamine state and neuropsychiatric syndromes: a systematic review and meta-analysis. https://doi.org/10.1136/jnnp-2021-327762. *J Neurol Neurosurg Psychiatry*. 2022;93(5):555-562.
71. Halahakoon DC, Kieslich K, O'Driscoll C, Nair A, Lewis G, Roiser JP. Reward-Processing Behavior in Depressed Participants Relative to Healthy Volunteers: A Systematic Review and Meta-analysis. https://doi.org/10.1001/jamapsychiatry.2020.2139. *JAMA Psychiatry*. 2020;77(12):1286-1295.
72. Addicott MA, Pearson JM, Schechter JC, Sapyta JJ, Weiss MD, Kollins SH. Attention-deficit/hyperactivity disorder and the explore/exploit trade-off. https://doi.org/10.1038/s41386-020-00881-8. *Neuropsychopharmacology*. 2021;46(3):614-621.
73. Admon R, Kaiser RH, Dillon DG, et al. Dopaminergic Enhancement of Striatal Response to Reward in Major Depression. https://doi.org/10.1176/appi.ajp.2016.16010111. *Am J Psychiatry*. 2017;174(4):378-386.
74. Chakroun K, Wiehler A, Wagner B, et al. Dopamine regulates decision thresholds in human reinforcement learning in males. https://doi.org/10.1038/s41467-023-41130-y. *Nat Commun*. 2023;14(1):5369-5369.
75. Cremer A, Kalbe F, Müller JC, Wiedemann K, Schwabe L. Disentangling the roles of dopamine and noradrenaline in the exploration-exploitation tradeoff during human decision-making. https://doi.org/10.1038/s41386-022-01517-9. *Neuropsychopharmacology*. 2023;48(7):1078-1086.
76. Diederen KM, Ziauddeen H, Vestergaard MD, Spencer T, Schultz W, Fletcher PC. Dopamine Modulates Adaptive Prediction Error Coding in the Human Midbrain and Striatum. https://doi.org/10.1523/jneurosci.1979-16.2016. *J Neurosci*. 2017;37(7):1708-1720.
77. Grob S, Pizzagalli DA, Dutra SJ, et al. Dopamine-related deficit in reward learning after catecholamine depletion in unmedicated, remitted subjects with bulimia nervosa. https://doi.org/10.1038/npp.2012.41. *Neuropsychopharmacology*. 2012;37(8):1945-52.
78. Haarsma J, Fletcher PC, Griffin JD, et al. Precision weighting of cortical unsigned prediction error signals benefits learning, is mediated by dopamine, and is impaired in psychosis. https://doi.org/10.1038/s41380-020-0803-8. *Mol Psychiatry*. 2021;26(9):5320-5333.
79. Halahakoon DC, Kaltenboeck A, Martens M, et al. Pramipexole Enhances Reward Learning by Preserving Value Estimates. https://doi.org/10.1016/j.biopsych.2023.05.023. *Biol Psychiatry*. 2024;95(3):286-296.
80. Howlett JR, Huang H, Hysek CM, Paulus MP. The effect of single-dose methylphenidate on the rate of error-driven learning in healthy males: a randomized controlled trial. https://doi.org/10.1007/s00213-017-4723-5. *Psychopharmacology (Berl)*. 2017;234(22):3353-3360.
81. Jansen M, Lockwood PL, Cutler J, de Bruijn ERA. l-DOPA and oxytocin influence the neurocomputational mechanisms of self-benefitting and prosocial reinforcement learning. https://doi.org/10.1016/j.neuroimage.2023.119983. *Neuroimage*. 2023;270:119983-119983.

82. Kahnt T, Weber SC, Haker H, Robbins TW, Tobler PN. Dopamine D2-receptor blockade enhances decoding of prefrontal signals in humans. https://doi.org/10.1523/jneurosci.4182-14.2015. *J Neurosci*. 2015;35(9):4104-11.

83. Murray GK, Knolle F, Ersche KD, et al. Dopaminergic drug treatment remediates exaggerated cingulate prediction error responses in obsessive-compulsive disorder. *Psychopharmacology*. 2019;236:2325-2336.

84. Walsh AEL, Huneke NTM, Brown R, Browning M, Cowen P, Harmer CJ. A Dissociation of the Acute Effects of Bupropion on Positive Emotional Processing and Reward Processing in Healthy Volunteers. https://doi.org/10.3389/fpsyt.2018.00482. *Front Psychiatry*. 2018;9:482-482.

85. Weis T, Puschmann S, Brechmann A, Thiel CM. Effects of L-dopa during auditory instrumental learning in humans. https://doi.org/10.1371/journal.pone.0052504. *PLoS One*. 2012;7(12):e52504-e52504.

86. Kelm MK, Boettiger CA. Age moderates the effect of acute dopamine depletion on passive avoidance learning. https://doi.org/10.1016/j.pbb.2015.01.014. *Pharmacol Biochem Behav*. 2015;131:57-63.

87. Daood M, Peled-Avron L, Ben-Hayun R, et al. Fronto-striatal connectivity patterns account for the impact of methylphenidate on choice impulsivity among healthy adults. https://doi.org/10.1016/j.neuropharm.2022.109190. *Neuropharmacology*. 2022;216:109190-109190.

88. Erfanian Abdoust M, Froböse MI, Schnitzler A, Schreivogel E, Jocham G. Dopamine and acetylcholine have distinct roles in delay- and effort-based decision-making in humans. https://doi.org/10.1371/journal.pbio.3002714. *PLoS Biol*. 2024;22(7):e3002714-e3002714.

89. Kayser AS, Allen DC, Navarro-Cebrian A, Mitchell JM, Fields HL. Dopamine, corticostriatal connectivity, and intertemporal choice. https://doi.org/10.1523/jneurosci.1180-12.2012. *J Neurosci*. 2012;32(27):9402-9.

90. Kelm MK, Boettiger CA. Effects of acute dopamine precusor depletion on immediate reward selection bias and working memory depend on catechol-O-methyltransferase genotype. https://doi.org/10.1162/jocn_a_00464. *J Cogn Neurosci*. 2013;25(12):2061-71.

91. Smith E, Theis H, van Eimeren T, et al. Dopamine and temporal discounting: revisiting pharmacology and individual differences. https://doi.org/10.1101/2024.08.28.610170. *bioRxiv*. 2024:2024.08.28.610170-2024.08.28.610170.

92. Wagner B, Clos M, Sommer T, Peters J. Dopaminergic Modulation of Human Intertemporal Choice: A Diffusion Model Analysis Using the D2-Receptor Antagonist Haloperidol. https://doi.org/10.1523/jneurosci.0592-20.2020. *J Neurosci*. 2020;40(41):7936-7948.

93. de Wit H, Enggasser JL, Richards JB. Acute administration of d-amphetamine decreases impulsivity in healthy volunteers. https://doi.org/10.1016/s0893-133x(02)00343-3. *Neuropsychopharmacology*. 2002;27(5):813-25.

94. Au-Yeung SK, Halahakoon DC, Kaltenboeck A, Cowen P, Browning M, Manohar SG. The effects of pramipexole on motivational vigour during a saccade task: a placebo-controlled study in healthy adults. https://doi.org/10.1007/s00213-024-06567-z. *Psychopharmacology (Berl)*. 2024;241(7):1365-1375.

95. Beierholm U, Guitart-Masip M, Economides M, et al. Dopamine modulates reward-related vigor. https://doi.org/10.1038/npp.2013.48. *Neuropsychopharmacology*. 2013;38(8):1495-503.
96. Graf H, Wiegers M, Metzger CD, Walter M, Abler B. Differential Noradrenergic Modulation of Monetary Reward and Visual Erotic Stimulus Processing. https://doi.org/10.3389/fpsyt.2018.00346. *Front Psychiatry*. 2018;9:346-346.
97. Grimm O, Nägele M, Küpper-Tetzel L, de Greck M, Plichta M, Reif A. No effect of a dopaminergic modulation fMRI task by amisulpride and L-DOPA on reward anticipation in healthy volunteers. https://doi.org/10.1007/s00213-020-05693-8. *Psychopharmacology (Berl)*. 2021;238(5):1333-1342.
98. Ikeda Y, Funayama T, Tateno A, Fukayama H, Okubo Y, Suzuki H. Bupropion increases activation in nucleus accumbens during anticipation of monetary reward. https://doi.org/10.1007/s00213-019-05337-6. *Psychopharmacology (Berl)*. 2019;236(12):3655-3665.
99. Mehta MA, Swainson R, Ogilvie AD, Sahakian J, Robbins TW. Improved short-term spatial memory but impaired reversal learning following the dopamine D(2) agonist bromocriptine in human volunteers. https://doi.org/10.1007/s002130100851. *Psychopharmacology (Berl)*. 2001;159(1):10-20.
100. Mehta MA, Gumaste D, Montgomery AJ, McTavish SF, Grasby PM. The effects of acute tyrosine and phenylalanine depletion on spatial working memory and planning in healthy volunteers are predicted by changes in striatal dopamine levels. https://doi.org/10.1007/s00213-004-2128-8. *Psychopharmacology (Berl)*. 2005;180(4):654-63.
101. Clatworthy PL, Lewis SJ, Brichard L, et al. Dopamine release in dissociable striatal subregions predicts the different effects of oral methylphenidate on reversal learning and spatial working memory. https://doi.org/10.1523/jneurosci.3266-08.2009. *J Neurosci*. 2009;29(15):4690-6.
102. Ersche KD, Roiser JP, Abbott S, et al. Response perseveration in stimulant dependence is associated with striatal dysfunction and can be ameliorated by a D(2/3) receptor agonist. https://doi.org/10.1016/j.biopsych.2011.06.033. *Biol Psychiatry*. 2011;70(8):754-762.
103. Janssen LK, Sescousse G, Hashemi MM, et al. Abnormal modulation of reward versus punishment learning by a dopamine D2-receptor antagonist in pathological gamblers. https://doi.org/10.1007/s00213-015-3986-y. *Psychopharmacology (Berl)*. 2015;232(18):3345-53.
104. Vo A, Seergobin KN, MacDonald PA. Independent effects of age and levodopa on reversal learning in healthy volunteers. https://doi.org/10.1016/j.neurobiolaging.2018.05.014. *Neurobiol Aging*. 2018;69:129-139.
105. van den Bosch R, Lambregts B, Määttä J, et al. Striatal dopamine dissociates methylphenidate effects on value-based versus surprise-based reversal learning. https://doi.org/10.1038/s41467-022-32679-1. *Nat Commun*. 2022;13(1):4962-4962.
106. van der Schaaf ME, Fallon SJ, Ter Huurne N, Buitelaar J, Cools R. Working memory capacity predicts effects of methylphenidate on reversal learning. *Neuropsychopharmacology : official publication of the American College of Neuropsychopharmacology*. 2013;38:2011-8.

107. van der Schaaf ME, van Schouwenburg MR, Geurts DEM, et al. Establishing the dopamine dependency of human striatal signals during reward and punishment reversal learning. *Cerebral cortex (New York, NY : 1991)*. 2014;24:633-42.

108. Campbell-Meiklejohn D, Wakeley J, Herbert V, et al. Serotonin and dopamine play complementary roles in gambling to recover losses. https://doi.org/10.1038/npp.2010.170. *Neuropsychopharmacology*. 2011;36(2):402-10.

109. Hirschbichler ST, Rothwell JC, Manohar SG. Dopamine increases risky choice while D2 blockade shortens decision time. https://doi.org/10.1007/s00221-022-06501-9. *Exp Brain Res*. 2022;240(12):3351-3360.

110. Norbury A, Manohar S, Rogers RD, Husain M. Dopamine modulates risk-taking as a function of baseline sensation-seeking trait. *The Journal of neuroscience : the official journal of the Society for Neuroscience*. 2013;33:12982-6.

111. Riba J, Krämer UM, Heldmann M, Richter S, Münte TF. Dopamine agonist increases risk taking but blunts reward-related brain activity. https://doi.org/10.1371/journal.pone.0002479. *PLoS One*. 2008;3(6):e2479-e2479.

112. Rigoli F, Rutledge RB, Chew B, Ousdal OT, Dayan P, Dolan RJ. Dopamine Increases a Value-Independent Gambling Propensity. https://doi.org/10.1038/npp.2016.68. *Neuropsychopharmacology*. 2016;41(11):2658-67.

113. Rutledge RB, Skandali N, Dayan P, Dolan RJ. Dopaminergic Modulation of Decision Making and Subjective Well-Being. https://doi.org/10.1523/jneurosci.0702-15.2015. *J Neurosci*. 2015;35(27):9811-22.

114. Colwell MJ, Tagomori H, Shang F, et al. Direct serotonin release in humans shapes aversive learning and inhibition. https://doi.org/10.1038/s41467-024-50394-x. *Nat Commun*. 2024;15(1):6617-6617.

115. Finger EC, Marsh AA, Buzas B, et al. The impact of tryptophan depletion and 5-HTTLPR genotype on passive avoidance and response reversal instrumental learning tasks. https://doi.org/10.1038/sj.npp.1301182. *Neuropsychopharmacology*. 2007;32(1):206-15.

116. Gilger MD, Hellrung L, Neukam PT, et al. Arbitration between model-free and model-based control is not affected by transient changes in tonic serotonin levels. https://doi.org/10.1177/02698811231216325. *J Psychopharmacol*. 2024;38(2):178-187.

117. Jepma M, Te Beek ET, Wagenmakers EJ, van Gerven JM, Nieuwenhuis S. The role of the noradrenergic system in the exploration-exploitation trade-off: a psychopharmacological study. https://doi.org/10.3389/fnhum.2010.00170. *Front Hum Neurosci*. 2010;4:170-170.

118. Michely J, Eldar E, Erdman A, Martin IM, Dolan RJ. Serotonin modulates asymmetric learning from reward and punishment in healthy human volunteers. https://doi.org/10.1038/s42003-022-03690-5. *Commun Biol*. 2022;5(1):812-812.

119. Gaber TJ, Dingerkus VL, Crockett MJ, et al. Studying the effects of dietary body weight-adjusted acute tryptophan depletion on punishment-related behavioral inhibition. https://doi.org/10.3402/fnr.v59.28443. *Food Nutr Res*. 2015;59:28443-28443.

120. Helmbold K, Zvyagintsev M, Dahmen B, et al. Effects of serotonin depletion on punishment processing in the orbitofrontal and anterior cingulate cortices of healthy women. https://doi.org/10.1016/j.euroneuro.2015.02.007. *Eur Neuropsychopharmacol*. 2015;25(6):846-56.

121. Crean J, Richards JB, de Wit H. Effect of tryptophan depletion on impulsive behavior in men with or without a family history of alcoholism. https://doi.org/10.1016/s0166-4328(02)00132-8. *Behav Brain Res*. 2002;136(2):349-57.

122. Schweighofer N, Bertin M, Shishida K, et al. Low-serotonin levels increase delayed reward discounting in humans. https://doi.org/10.1523/jneurosci.4982-07.2008. *J Neurosci*. 2008;28(17):4528-32.

123. Tanaka SC, Schweighofer N, Asahi S, et al. Serotonin differentially regulates short- and long-term prediction of rewards in the ventral and dorsal striatum. https://doi.org/10.1371/journal.pone.0001333. *PLoS One*. 2007;2(12):e1333-e1333.

124. Worbe Y, Savulich G, Voon V, Fernandez-Egea E, Robbins TW. Serotonin depletion induces 'waiting impulsivity' on the human four-choice serial reaction time task: cross-species translational significance. *Neuropsychopharmacology : official publication of the American College of Neuropsychopharmacology*. 2014;39:1519-26.

125. Cools R, Blackwell A, Clark L, Menzies L, Cox S, Robbins TW. Tryptophan depletion disrupts the motivational guidance of goal-directed behavior as a function of trait impulsivity. https://doi.org/10.1038/sj.npp.1300704. *Neuropsychopharmacology*. 2005;30(7):1362-73.

126. Roiser JP, Blackwell AD, Cools R, et al. Serotonin transporter polymorphism mediates vulnerability to loss of incentive motivation following acute tryptophan depletion. https://doi.org/10.1038/sj.npp.1301055. *Neuropsychopharmacology*. 2006;31(10):2264-72.

127. Steding J, Ritschel F, Boehm I, et al. The effects of acute tryptophan depletion on instrumental reward learning in anorexia nervosa - an fMRI study. https://doi.org/10.1017/s0033291721005493. *Psychol Med*. 2023;53(8):3426-3436.

128. Chamberlain SR, Müller U, Blackwell AD, Clark L, Robbins TW, Sahakian BJ. Neurochemical modulation of response inhibition and probabilistic learning in humans. https://doi.org/10.1126/science.1121218. *Science*. 2006;311(5762):861-3.

129. Evers EAT, Tillie DE, van der Veen FM, et al. Effects of a novel method of acute tryptophan depletion on plasma tryptophan and cognitive performance in healthy volunteers. https://doi.org/10.1007/s00213-004-1933-4. *Psychopharmacology*. 2004;177(1):217–223-217–223.

130. Kanen JW, Arntz FE, Yellowlees R, et al. Probabilistic reversal learning under acute tryptophan depletion in healthy humans: a conventional analysis. https://doi.org/10.1177/0269881120907991. *J Psychopharmacol*. 2020;34(5):580-583.

131. Langley C, Armand S, Luo Q, et al. Chronic escitalopram in healthy volunteers has specific effects on reinforcement sensitivity: a double-blind, placebo-controlled semi-randomised study. https://doi.org/10.1038/s41386-022-01523-x. *Neuropsychopharmacology*. 2023;48(4):664-670.

132. Skandali N, Rowe JB, Voon V, et al. Dissociable effects of acute SSRI (escitalopram) on executive, learning and emotional functions in healthy humans. https://doi.org/10.1038/s41386-018-0229-z. *Neuropsychopharmacology*. 2018;43(13):2645-2651.

133. Thirkettle M, Barker LM, Gallagher T, Nayeb N, Aquili L. Dissociable Effects of Tryptophan Supplementation on Negative Feedback Sensitivity and Reversal Learning. https://doi.org/10.3389/fnbeh.2019.00127. *Front Behav Neurosci*. 2019;13:127-127.

134. Worbe Y, Savulich G, de Wit S, Fernandez-Egea E, Robbins TW. Tryptophan Depletion Promotes Habitual over Goal-Directed Control of Appetitive Responding in Humans. https://doi.org/10.1093/ijnp/pyv013. *Int J Neuropsychopharmacol*. 2015;18(10):pyv013-pyv013.

135. Anderson IM, Richell RA, Bradshaw CM. The effect of acute tryptophan depletion on probabilistic choice. https://doi.org/10.1177/0269881103017001687. *J Psychopharmacol*. 2003;17(1):3-7.

136. Faulkner P, Mancinelli F, Lockwood PL, et al. Peripheral Serotonin 1B Receptor Transcription Predicts the Effect of Acute Tryptophan Depletion on Risky Decision-Making. *The international journal of neuropsychopharmacology*. 2017;20:58-66.

137. Macoveanu J, Fisher PM, Haahr ME, Frokjaer VG, Knudsen GM, Siebner HR. Effects of selective serotonin reuptake inhibition on neural activity related to risky decisions and monetary rewards in healthy males. https://doi.org/10.1016/j.neuroimage.2014.05.040. *Neuroimage*. 2014;99:434-42.

138. Murphy SE, Longhitano C, Ayres RE, Cowen PJ, Harmer CJ, Rogers RD. The role of serotonin in nonnormative risky choice: the effects of tryptophan supplements on the "reflection effect" in healthy adult volunteers. https://doi.org/10.1162/jocn.2009.21122. *J Cogn Neurosci*. 2009;21(9):1709-19.

4 eAppendix 1

4.1 PRISMA 2020 Checklist

Section and Topic	Item #	Checklist item	Location where item is reported
**TITLE**			
Title	1	Identify the report as a systematic review.	Title
**ABSTRACT**			
Abstract	2	See the PRISMA 2020 for Abstracts checklist.	Appendix 1: 4.2 PRISMA 2020 Abstract Checklist
**INTRODUCTION**			
Rationale	3	Describe the rationale for the review in the context of existing knowledge.	Introduction
Objectives	4	Provide an explicit statement of the objective(s) or question(s) the review addresses.	Introduction
**METHODS**			
Eligibility criteria	5	Specify the inclusion and exclusion criteria for the review and how studies were grouped for the syntheses.	Methods, Supplementary materials: 1.2 Paper selection, 1.3 Meta-analysis
Information sources	6	Specify all databases, registers, websites, organisations, reference lists and other sources searched or consulted to identify studies. Specify the date when each source was last searched or consulted.	Methods
Search strategy	7	Present the full search strategies for all databases, registers and websites, including any filters and limits used.	eTable 1
Selection process	8	Specify the methods used to decide whether a study met the inclusion criteria of the review, including how many reviewers screened each record and each report retrieved, whether they worked independently, and if applicable, details of automation tools used in the process.	Supplementary methods: 1.2 Paper selection
Data collection process	9	Specify the methods used to collect data from reports, including how many reviewers collected data from each report, whether they worked independently, any processes for obtaining or confirming data from study investigators, and if applicable, details of automation tools used in the process.	Supplementary methods: 1.2 Paper selection, 1.3 Meta-analysis
Data items	10a	List and define all outcomes for which data were sought. Specify whether all results that were compatible with each outcome domain in each study were sought (e.g. for all measures, time points, analyses), and if not, the methods used to decide which results to collect.	Methods, Supplementary methods: 1.2 Paper selection, 1.3 Meta-analysis
	10b	List and define all other variables for which data were sought (e.g. participant and intervention characteristics, funding sources). Describe any assumptions made about any missing or unclear information.	eTable5-6
Study risk of bias assessment	11	Specify the methods used to assess risk of bias in the included studies, including details of the tool(s) used, how many reviewers assessed each study and whether they worked independently, and if applicable, details of automation tools used in the process.	Supplementary methods: 1.3.5 Assessment of study quality
Effect measures	12	Specify for each outcome the effect measure(s) (e.g. risk ratio, mean difference) used in the synthesis or presentation of results.	Methods

Section and Topic	Item #	Checklist item	Location where item is reported
Synthesis methods	13a	Describe the processes used to decide which studies were eligible for each synthesis (e.g. tabulating the study intervention characteristics and comparing against the planned groups for each synthesis (item #5)).	Methods, Supplementary methods: 1.2 Paper selection, 1.3 Meta-analysis
	13b	Describe any methods required to prepare the data for presentation or synthesis, such as handling of missing summary statistics, or data conversions.	Supplementary methods: 1.4 Effect size calculations
	13c	Describe any methods used to tabulate or visually display results of individual studies and syntheses.	Figure 2-5, eFigure 3-13
	13d	Describe any methods used to synthesize results and provide a rationale for the choice(s). If meta-analysis was performed, describe the model(s), method(s) to identify the presence and extent of statistical heterogeneity, and software package(s) used.	Methods, Supplementary methods: 1.3 Meta-analysis
	13e	Describe any methods used to explore possible causes of heterogeneity among study results (e.g. subgroup analysis, meta-regression).	Supplementary methods: 1.3 Meta-analysis
	13f	Describe any sensitivity analyses conducted to assess robustness of the synthesized results.	Methods, Supplementary methods: 1.3 Meta-analysis
Reporting bias assessment	14	Describe any methods used to assess risk of bias due to missing results in a synthesis (arising from reporting biases).	Supplementary methods: 1.3 Meta-analysis
Certainty assessment	15	Describe any methods used to assess certainty (or confidence) in the body of evidence for an outcome.	Supplementary methods: 1.3.5 Assessment of study quality
**RESULTS**			
Study selection	16a	Describe the results of the search and selection process, from the number of records identified in the search to the number of studies included in the review, ideally using a flow diagram.	Figure 1
	16b	Cite studies that might appear to meet the inclusion criteria, but which were excluded, and explain why they were excluded.	Supplementary methods: 1.2 Paper selection, Figure 1
Study characteristics	17	Cite each included study and present its characteristics.	Figure 2-5, eFigure 3-13, eTable 5-6
Risk of bias in studies	18	Present assessments of risk of bias for each included study.	eFigure 2
Results of individual studies	19	For all outcomes, present, for each study: (a) summary statistics for each group (where appropriate) and (b) an effect estimate and its precision (e.g. confidence/credible interval), ideally using structured tables or plots.	Figure 2-5, eFigure 3-13
Results of syntheses	20a	For each synthesis, briefly summarise the characteristics and risk of bias among contributing studies.	Results
	20b	Present results of all statistical syntheses conducted. If meta-analysis was done, present for each the summary estimate and its precision (e.g. confidence/credible interval) and measures of statistical heterogeneity. If comparing groups, describe the direction of the effect.	Results, Figure 2-5, eFigure 3-13, Supplementary results

Section and Topic	Item #	Checklist item	Location where item is reported
	20c	Present results of all investigations of possible causes of heterogeneity among study results.	Results, Supplementary results: 2.3 Dopamine heterogeneity, 2.5 serotonin heterogeneity, 2.6 Moderator analyses, 2.7 Publication bias
	20d	Present results of all sensitivity analyses conducted to assess the robustness of the synthesized results.	Supplementary results: 2.2 Additional dopamine results, 2.4 Additional serotonin results, 2.8 Additional supplementary analyses
Reporting biases	21	Present assessments of risk of bias due to missing results (arising from reporting biases) for each synthesis assessed.	Results, Supplementary methods: 2.7 Publication bias
Certainty of evidence	22	Present assessments of certainty (or confidence) in the body of evidence for each outcome assessed.	Results, Figures 2-5, eFigure 3-13
**DISCUSSION**			
Discussion	23a	Provide a general interpretation of the results in the context of other evidence.	Discussion
	23b	Discuss any limitations of the evidence included in the review.	Discussion
	23c	Discuss any limitations of the review processes used.	Discussion
	23d	Discuss implications of the results for practice, policy, and future research.	Discussion
**OTHER INFORMATION**			
Registration and protocol	24a	Provide registration information for the review, including register name and registration number, or state that the review was not registered.	Methods, Additional information
	24b	Indicate where the review protocol can be accessed, or state that a protocol was not prepared.	Additional information
	24c	Describe and explain any amendments to information provided at registration or in the protocol.	In the preregistered protocol
Support	25	Describe sources of financial or non-financial support for the review, and the role of the funders or sponsors in the review.	Financial disclosures
Competing interests	26	Declare any competing interests of review authors.	Acknowledgments
Availability of data, code and other materials	27	Report which of the following are publicly available and where they can be found: template data collection forms; data extracted from included studies; data used for all analyses; analytic code; any other materials used in the review.	Additional information

4.2 PRISMA 2020 Abstract Checklist

Section and Topic	Item #	Checklist item	Reported (Yes/No)
**TITLE**			
Title	1	Identify the report as a systematic review.	Yes
**BACKGROUND**			
Objectives	2	Provide an explicit statement of the main objective(s) or question(s) the review addresses.	Yes
**METHODS**			
Eligibility criteria	3	Specify the inclusion and exclusion criteria for the review.	Yes
Information sources	4	Specify the information sources (e.g. databases, registers) used to identify studies and the date when each was last searched.	Yes
Risk of bias	5	Specify the methods used to assess risk of bias in the included studies.	Yes
Synthesis of results	6	Specify the methods used to present and synthesise results.	Yes
**RESULTS**			
Included studies	7	Give the total number of included studies and participants and summarise relevant characteristics of studies.	Yes
Synthesis of results	8	Present results for main outcomes, preferably indicating the number of included studies and participants for each. If meta-analysis was done, report the summary estimate and confidence/credible interval. If comparing groups, indicate the direction of the effect (i.e. which group is favoured).	Yes
**DISCUSSION**			
Limitations of evidence	9	Provide a brief summary of the limitations of the evidence included in the review (e.g. study risk of bias, inconsistency and imprecision).	No
Interpretation	10	Provide a general interpretation of the results and important implications.	Yes
**OTHER**			
Funding	11	Specify the primary source of funding for the review.	No
Registration	12	Provide the register name and registration number.	No
